# Machine learning Hubbard parameters with equivariant neural networks

**DOI:** 10.1038/s41524-024-01501-5

**Published:** 2025-01-25

**Authors:** Martin Uhrin, Austin Zadoks, Luca Binci, Nicola Marzari, Iurii Timrov

**Affiliations:** 1 Theory and Simulation of Materials (THEOS), and National Centre for Computational Design and Discovery of Novel Materials (MARVEL), École Polytechnique Fédérale de Lausanne (EPFL), Lausanne, Switzerland; 2 Université Grenoble Alpes, St Martin D’Heres, France; 3 Laboratory for Materials Simulations (LMS), Paul Scherrer Institut (PSI), Villigen, Switzerland; 4 Present Address: Department of Materials Science & Engineering, University of California Berkeley, Berkeley, CA USA

**Keywords:** Computational methods, Electronic properties and materials, Theoretical chemistry

## Abstract

Density-functional theory with extended Hubbard functionals (DFT + *U* + *V*) provides a robust framework to accurately describe complex materials containing transition-metal or rare-earth elements. It does so by mitigating self-interaction errors inherent to semi-local functionals which are particularly pronounced in systems with partially-filled d and f electronic states. However, achieving accuracy in this approach hinges upon the accurate determination of the on-site *U* and inter-site *V* Hubbard parameters. In practice, these are obtained either by semi-empirical tuning, requiring prior knowledge, or, more correctly, by using predictive but expensive first-principles calculations. Here, we present a machine learning model based on equivariant neural networks which uses atomic occupation matrices as descriptors, directly capturing the electronic structure, local chemical environment, and oxidation states of the system at hand. We target here the prediction of Hubbard parameters computed self-consistently with iterative linear-response calculations, as implemented in density-functional perturbation theory (DFPT), and structural relaxations. Remarkably, when trained on data from 12 materials spanning various crystal structures and compositions, our model achieves mean absolute relative errors of 3% and 5% for Hubbard *U* and *V* parameters, respectively. By circumventing computationally expensive DFT or DFPT self-consistent protocols, our model significantly expedites the prediction of Hubbard parameters with negligible computational overhead, while approaching the accuracy of DFPT. Moreover, owing to its robust transferability, the model facilitates accelerated materials discovery and design via high-throughput calculations, with relevance for various technological applications.

## Introduction

A fundamental tool in investigating compounds involving transition-metal (TM) and rare-earth (RE) compounds is density-functional theory (DFT)^1,2^, a cornerstone for first-principles simulations in physics, chemistry, and materials science. In practical applications, DFT necessitates approximations to the exchange-correlation functional, with the local spin-density approximation and spin-polarized generalized-gradient approximation (*σ*-GGA) being the most prevalent choices. However, these approximations yield unsatisfactory outcomes for various properties of TM and RE compounds, primarily due to significant self-interaction errors (SIEs)^3–5^ that are particularly pronounced for localized d and f electrons. To address these challenges, more accurate approaches surpassing the limitations of “standard DFT” have been devised. Noteworthy among these are Hubbard-corrected DFT (so-called DFT + *U*^6–8^ and its extension DFT + *U* + *V*^9–11^, whose role in addressing SIEs, rather than correlation errors, was first pointed out in ref. ^4^), meta-GGA functionals^12–14^, and hybrid functionals^15–17^. While these methods offer valuable insights, each comes with inherent limitations and challenges, as discussed in, e.g., ref. ^18^. Hubbard-corrected DFT in particular stands out for its greater accuracy with only a marginal increase in computational cost over standard DFT functionals^19^. In the DFT + *U* + *V* scheme, a corrective Hubbard energy *E*_*U*+*V*_ is introduced alongside the approximate DFT energy *E*_DFT_:

1 $${E}_{{{\rm{DFT}}}+U+V}={E}_{{{\rm{DFT}}}}+{E}_{U+V}.$$

In the simplified rotationally-invariant formulation^8^, the extended Hubbard correction energy for a manifold with angular momentum *ℓ* takes the form^20^:

2 $$\begin{array}{ll}{E}_{U+V}\,=\,\frac{1}{2}\sum\limits_{I\sigma }{U}^{I}{{\rm{Tr}}}\left[{{{\bf{n}}}}_{\ell }^{II\sigma }\left({{\bf{1}}}-{{{\bf{n}}}}_{\ell }^{II\sigma }\right)\right]\\ \qquad\qquad\,\,-\frac{1}{2}\sum\limits_{I}\sum\limits_{J(J\ne I)}^{* }\sum\limits_{\sigma }{V}^{IJ}{{\rm{Tr}}}\left[{{{\bf{n}}}}_{\ell }^{IJ\sigma }{{{\bf{n}}}}_{\ell }^{JI\sigma }\right],\end{array}$$

where *I* and *J* are atomic site indices, and *σ* is the spin index. *U*^*I*^ and *V*^*IJ*^ are effective on-site and inter-site Hubbard parameters, respectively. The asterisk in the sum signifies that, for each atom *I*, the index *J* encompasses all its neighbors up to a given distance. The generalized occupation matrices ${{{\bf{n}}}}_{\ell }^{IJ\sigma }$ are derived from the projection of the Kohn-Sham (KS) states onto localized atom-centered orbitals ${\phi }_{m}^{I}({{\bf{r}}})$ (Hubbard projector functions) of neighboring atoms:

3 $${{{\bf{n}}}}_{\ell }^{IJ\sigma }\equiv {n}_{m{m}^{{\prime} }}^{IJ\sigma }=\sum\limits_{v,{{\bf{k}}}}{f}_{v,{{\bf{k}}}}^{\sigma }\langle {\psi }_{v,{{\bf{k}}}}^{\sigma }| {\phi }_{{m}^{{\prime} }}^{J}\rangle \langle {\phi }_{m}^{I}| {\psi }_{v,{{\bf{k}}}}^{\sigma }\rangle ,$$

where *v* represents the band index of the KS wavefunctions ${\psi }_{v,{{\bf{k}}}}^{\sigma }({{\bf{r}}})$, *m* and ${m}^{{\prime} }$ denote magnetic quantum numbers associated with a specific angular momentum ℓ, **k** denotes points in the first Brillouin zone (BZ), and ${f}_{v,{{\bf{k}}}}^{\sigma }$ are the occupations of the KS states. The two terms in eq. (2)—proportional to on-site *U*^*I*^ and inter-site *V*^*IJ*^—counteract one another. The on-site term promotes localization on atomic sites, suppressing hybridization with neighbors, while the inter-site term favors hybridized states with components on neighboring atoms. Consequently, the values of *U*^*I*^ and *V*^*IJ*^ are critical to optimizing the extent of the localization and hybridization within Hubbard-corrected DFT. However, these parameters are not known a priori and must be determined in some way. In passing we note that for the sake of simplicity, hereafter we drop the superscripts *I* and *J* in the notations of Hubbard parameters and occupation matrices, unless required for clarity.

Hubbard *U* can be fit semi-empirically to reproduce a target property from experimental data^21–25^ or from other advanced first-principles methods (e.g., *GW*^26^ or hybrids^15–17^); however, this approach has many limitations. Fitted parameters are not guaranteed to, and often do not, accurately predict properties other than those used for fitting, and calibrating a single Hubbard *U* to multiple properties is non-trivial, requiring advanced algorithms like Bayesian optimization (BO)^27,28^. This method also precludes materials discovery, where properties are by definition unknown, and targeting results of advanced first-principles methods, like DFT with hybrid functionals, inherits the limitations of those methods^29,30^. Moreover, the inter-site *V* parameters, which are necessary to properly describe materials with pronounced covalent interactions, are difficult to fit semi-empirically due to the high-dimensional regression procedure they would require. An attractive alternative is computing Hubbard parameters using first-principles methods such as constrained DFT (cDFT)^31–39^, Hartree-Fock-based approaches^10,11,40–43^, and the constrained random phase approximation^44–47^. These methods do not rely on data from experiments or advanced simulation methods and, moreover, are able to provide values not only for *U* but also for *V*. However, first-principles approaches are considerably more computationally expensive than DFT + *U*(+*V*) ground-state calculations themselves, making their application feasible but demanding for large systems or high-throughput studies.

**Fig. 1:**
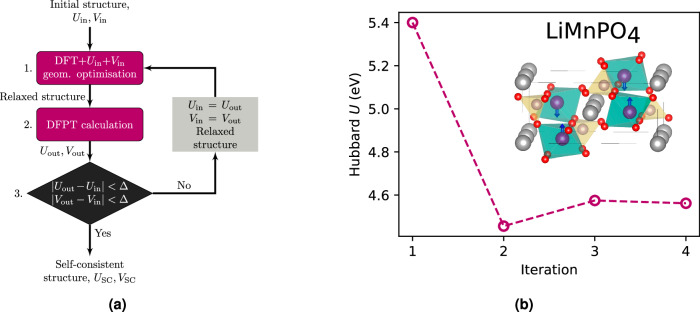
Calculating Hubbard corrections self-consistently using density-functional perturbation theory. **a** Protocol for the self-consistent calculation of Hubbard parameters using DFPT^49^. *U*_in_ and *V*_in_ represent the input Hubbard parameters, while *U*_out_ and *V*_out_ denote the output parameters, with *Δ* representing the convergence threshold. *U*_SC_ and *V*_SC_ are the final SC Hubbard parameters. **b** Convergence of the Hubbard *U* parameter for Mn-3d states in LiMnPO_4_ using the self-consistent protocol^18^. The inset displays the crystal structure of the material, where arrows indicate the spin direction, and Li atoms are depicted in gray, O in red, Mn in violet, and P in yellow.

The linear-response (LR) formulation of cDFT (LR-cDFT)^20^ has witnessed popularity due to its simplicity and accuracy; however, it demands computationally expensive supercell calculations. A recent reformulation of LR-cDFT in terms of density-functional perturbation theory (DFPT)^48,49^ significantly reduces the computational burden for determining Hubbard parameters by replacing cumbersome supercell calculations with faster unit-cell calculations and by leveraging symmetries that further diminishes the computational cost by reducing the number of perturbations in reciprocal space (see Sec. S1 in the Supplemental Information (SI)). Its physical rationale relies on heuristically imposing piecewise linearity of the total energy of the system as a function of the population of the Hubbard manifold^20^. Despite the numerous successful applications of DFPT in computing Hubbard parameters^18,50–57^, this approach introduces a significant overhead compared to DFT + *U* + *V* ground-state calculations. Moreover, it has been shown that jointly optimizing Hubbard parameters and the crystal structure, rather than relying on the equilibrium geometry obtained using (semi-)local functionals, can significantly improve the accuracy of the final properties of interest^58^. This is due in part to taking into account the geometry dependence of *U*{**R**} and thus the contribution *dU*/*d***R** to the Hellmann-Feynman forces^59^. To do so, a self-consistent (SC) procedure combining DFPT and structural optimizations (Fig. 1) can be used (see Sec. S2 in the SI)^49,60^. Figure 1 shows a typical SC determination of Hubbard parameters for LiMnPO_4_ following this procedure, where each iteration costs approximately one order of magnitude more computational time than a Hubbard-corrected DFT electronic ground-state calculation. Consequently, the adoption of acceleration techniques is highly sought after to expedite the determination of Hubbard parameters while providing a level of accuracy very close to that of DFPT and, prospectively, a simple estimate of *dU*/*d***R** and *dV*/*d***R**.

Using machine learning (ML) to accelerate parts of a DFT workflow is becoming increasingly common^61,62^, including applications to predicting Hubbard parameters. A recent example is the work of Cai et al. ^63^, in which they use crystal structure parameters, such as bond lengths and angles (among others), as descriptors for a random forest regression model trained on a database of Mn oxides. Their training data consist of *U* values fitted using BO to replicate band gaps and band structures obtained from HSE hybrid functional calculations—a concept initially proposed in the earlier work of ref. ^27^ within the context of DFT + *U* and subsequently extended to the DFT + *U* + *V* framework^64^. The resulting ML model predicts *U* values such that subsequent DFT + *U* calculations using these values yield band gaps that are 2–3 times smaller than the reference HSE values, while the band structures appear qualitatively similar. Despite the significance of these efforts in applying ML to the prediction of Hubbard parameters, numerous critical issues persist, such as the accuracy of the reference training data (HSE often provides unreliable band gaps for solids^29,30^), the choice of the target properties for the training data (fitting *U* to reproduce band gaps is questionable), the importance of self-consistency between Hubbard parameters and the crystal structure, as well as the lack of inter-site *V*, to name a few. Addressing these points is highly relevant and necessitates further investigation, which serves as the motivation for this work.

Here, we introduce a novel ML approach based on equivariant neural-networks (ENNs) which aims to replace computationally demanding first-principles DFPT calculations of Hubbard *U* and *V* parameters while providing a negligible loss of accuracy for the vast majority of practical applications. Crucially, the model employs: (1) atomic occupation matrices within the DFT + *U* + *V* framework as descriptors of the geometry, electronic structure (e.g., oxidation states (OSs)) and local chemical environments in materials, as well as (2) DFPT-based Hubbard parameters, and (3) interatomic distances. This model is general and can be applied to materials with ionic, covalent, and mixed ionic-covalent interactions. It is trained on all intermediate Hubbard parameters and occupation matrices obtained during the SC cycle and directly provides the final SC values of *U* and *V*, thus also bypassing intermediate structural optimizations. The utilization of ENNs facilitates the exploitation of the inherent O(3) group structure of the occupation matrices, ensuring excellent model performance even with scarce training data. Equivariant models have demonstrated state-of-the-art accuracy and transferability in ML interaction potentials^65,66^, while our work is the first to incorporate electronic-structure degrees of freedom as explicit features in solids.

## Results

### Occupation matrices as the model inputs

The goal of our ML approach is to replace the SC procedure represented in Fig. 1 and provide the final Hubbard parameters using as input results from an initial DFT(+*U* + *V*) calculation. Conventional ML methods for atomistic systems primarily use the ionic structures as inputs (see e.g., refs. ^67–70^), side-stepping the need to explicitly calculate or even consider the electronic structure. However, as a consequence such models may not be particularly sensitive to changes in electronic structure that only lead to subtle changes in local atomic geometry, as can happen during a change of OS. To overcome this limitation, our models take as input the on-site occupation matrices ${{{\bf{n}}}}_{\ell }^{\sigma }\equiv {{{\bf{n}}}}_{\ell }^{II\sigma }$ [see Eq. (3), *I* = *J*], which describe the local electronic structure around an atom, including the OS which is reflected in the occupation matrix eigenvalues^71^. This is a particularly compelling choice given the significant variations in Hubbard parameters seen for different OSs of TM elements^18,52^. We emphasize that relying solely on the trace of the occupation matrix as a descriptor may not adequately distinguish between different OSs and chemical environments.

As with any learning task, the ML model should respect the way in which the target physical properties transform under global rotations, translations, reflections and permutations of labels. In the present case, the output *U* and *V* values are left unchanged by these transformations, however the entries of the occupation matrix *do* change under rotation, making ${n}_{m{m}^{{\prime} }}^{\sigma }$ unsuitable for use as inputs to non-symmetry aware models. For this reason, we make use of an *equivariant* learning model^72–75^, which has the property that the learned function *f*: *X* → *Y* obeys the following relation:

4 $${D}_{Y}[g]f(x)=f({D}_{X}[g]x),\quad \forall g\in G,\,\,\forall x\in X,$$

where the equivariance is with respect to a group *G* (in our case SE (3)), and *D*_*Y*_[*g*] and *D*_*X*_[*g*] are representations that act on the vector spaces *Y* and *X*, respectively. For example, applying a rotation to the inputs and then applying *f* must produce the same outputs as applying the rotation to the outputs of *f* given the original (unrotated) input. While our model outputs scalars (which are symmetry invariant), by using an equivariant model we ensure that the inputs and all intermediate (hidden) features transform together under group actions. This property has been shown to give state-of-the-art accuracy and transferability, particularly within the domain of ML interaction potentials^65,70,76^.

### Equivariant descriptors

By virtue of being atom-centered, the on-site occupation matrix ${{{\bf{n}}}}_{\ell }^{\sigma }$ is naturally invariant to global translations. However, to build a rotationally invariant model, it is convenient to re-express the occupation matrix in terms of the irreducible representations (irreps) of the O(3) group. If we let *Γ*^(*ℓ*, *P*)^ be the irrep with degree *ℓ* and parity *P*, we can express the occupation matrices as the tensor products *Γ*^(*ℓ*, *P*)^ ⊗ *Γ*^(*ℓ*, *P*)^, where e.g., *ℓ* = 1 for p orbitals, and *ℓ* = 2 for d orbitals. These can then be decomposed into a direct sum of irreps, e.g., for *ℓ* = 1 and *P* = −1 the occupation matrix is a 3 × 3, rank-2, tensor *Γ*^(1, −1)^ ⊗ *Γ*^(1, −1)^, that is symmetric, as ${{{\bf{n}}}}_{\ell }^{\sigma }={\left({{{\bf{n}}}}_{\ell }^{\sigma }\right)}^{{\mathsf{T}}}$. In practice, this decomposition is achieved by applying a change of basis for ${{{\bf{n}}}}_{\ell }^{\sigma }$ that transforms it into the irrep basis (see Supplementary Section 3 for details). At this stage, the remaining symmetry to be addressed is that of label permutation. In the case of the atom labels for the inter-site term, the model should be invariant to permutation of *I* and *J*, however in our training data the Hubbard *V* correction is always applied to a d-block/p-block atom pairs which have occupation matrices of different dimension (5 × 5 for d and 3 × 3 for p), thereby making them distinguishable.

There remains a permutational invariance to be imposed on the spin labels (*σ* = *↑* or *↓*). We achieve this using permutationally invariant polynomials:

5 $${{{\bf{x}}}}_{\ell }^{1}={{{\bf{n}}}}_{\ell }^{\uparrow }+{{{\bf{n}}}}_{\ell }^{\downarrow },$$

6 $${{{\bf{x}}}}_{\ell }^{2}={{{\bf{n}}}}_{\ell }^{\uparrow }\otimes {{{\bf{n}}}}_{\ell }^{\downarrow }.$$

In the following, for the sake of convenience, we label these tensors in their irrep form as ${{{\bf{x}}}}_{{{\rm{p}}}}^{i}$ for p orbitals and ${{{\bf{x}}}}_{{{\rm{d}}}}^{i}$ for d orbitals, where *i* can be 1 or 2. We note in passing that in the non-spin-polarized case, one can set ${{{\bf{n}}}}_{\ell }^{\uparrow }={{{\bf{n}}}}_{\ell }^{\downarrow }={{{\bf{n}}}}_{\ell }/2$, where **n**_*ℓ*_ is divided by two to account for spin degeneracy, and then proceed as above. In addition, atomic species (i.e., atomic types) are encoded as one-hot vectors in our ML model. A summary of the irreps of all the model inputs is presented in Table 1.

**Table 1:** Irreducible representations of various attributes (inputs and outputs) of the ML model

Attribute	Irreducible representation
*U*^*I*^, *V*^*IJ*^, *r*_*IJ*_	*Γ*^(0, 1)^
Atomic species	${\bigoplus }^{{N}_{s}}{\Gamma }^{(0,1)}$
${{{\bf{n}}}}_{{{\rm{p}}}}^{\sigma }$	*Γ*^(0, 1)^ ⊕ *Γ*^(2, 1)^
${{{\bf{n}}}}_{{{\rm{d}}}}^{\sigma }$	*Γ*^(0, 1)^ ⊕ *Γ*^(2, 1)^ ⊕ *Γ*^(4, 1)^

*U*^*I*^ and *V*^*IJ*^ are the on-site and inter-site Hubbard parameters, respectively, *r*_*IJ*_ is the interatomic distance between species *I* and *J*, *N*_*s*_ is the number of atomic species (one-hot vectors), ${{{\bf{n}}}}_{{{\rm{p}}}}^{\sigma }$ and ${{{\bf{n}}}}_{{{\rm{d}}}}^{\sigma }$ are the occupation matrices for the p and d orbitals, respectively, in the special representation for the ML model [see Eqs. (5) and (6)]. The irreducible representations of the occupation matrices can be readily obtained using the e3nn^75,79^ library.

In the case of the ML model for Hubbard *V*, the interatomic distances *r*_*IJ*_ are also included as an additional input, giving some basic information on the ionic structure. It is important to note that we exclusively focus on the on-site occupation matrices ${{{\bf{n}}}}_{\ell }^{II\sigma }$ for practical reasons. While inter-site matrices (see, e.g.,^77,78^) could be naturally included in our models, Quantum ESPRESSO does not currently output these. Additionally, this approach ensures consistency with the LR approach used to calculate the Hubbard *V* parameters from first principles, which relies on the response of on-site occupation matrices only^9,49^. However, using inter-site occupation matrices ${{{\bf{n}}}}_{\ell }^{IJ\sigma }$ as descriptors instead of *r*_*IJ*_ presents an interesting alternative that could be explored in future studies. Finally, while additional descriptors, such as the derivatives of the occupation matrix with respect to the Hubbard parameters, could potentially enhance the performance of the ML model, this would significantly increase the cost of generating training data.

### Equivariant neural-network model

**Fig. 2:**
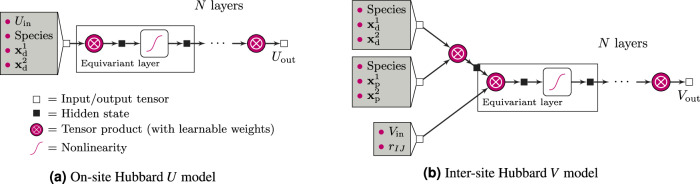
Schematic illustration of the equivariant neural-network ML model for predicting Hubbard parameters. **a** The model for predicting on-site *U* values, **b** the model for inter-site *V* values. *U*_in_ and *V*_in_ are the input Hubbard parameters, while *U*_out_ and *V*_out_ are the outputs. The atomic species enter as one-hot tensors, and *r*_*IJ*_ is the interatomic distance between sites *I* and *J*. ${{{\bf{x}}}}_{{{\rm{d}}}}^{1}$ and ${{{\bf{x}}}}_{{{\rm{d}}}}^{2}$ are the occupation matrices for the d orbitals, while ${{{\bf{x}}}}_{{{\rm{p}}}}^{1}$ and ${{{\bf{x}}}}_{{{\rm{p}}}}^{2}$ for the p orbitals, all in the special representation for the ML model [see Eqs. (5) and (6)]. Tensors are represented as squares: open for inputs and outputs, and filled for intermediate features.

To construct our ML model, we use the e3nn library^75,79^ and PyTorch^80^; our codebase is open-source and freely available (see “*code availability*” below). As shown in Fig. 2, we define two separate models, one for predicting the on-site *U* values and the other for the inter-site *V* ones. By using separate models, we provide flexibility for the model to be used in calculations where only Hubbard *U* is applied (i.e., DFT + *U*). In each case, the model starts with one or more nodes that carry attributes, expressed as a direct sum of irreps, which represent the inputs to the learned function. The inputs pass through a series of repeated layers made up of a tensor product (analogous to all-to-all connected layers in a traditional neural network) and a gated non-linearity (an equivariant version of a traditional activation function^79^) followed by a final tensor product before readout. For each experiment, we rescale the model outputs to have zero mean and a standard deviation of one based on the Hubbard parameters found in the training set which simultaneously accelerates training and improves the final loss. All of the tensor products have learnable weights, meaning that every pair of input irreps that contribute to one output irrep has a learnable scalar parameter that is optimized during training. We use the AdamW^81^ optimizer to minimize the loss, which is the mean squared error between predicted and training Hubbard parameters.

### Training and validation datasets

To train and validate our ENN model, we curated a dataset comprising materials with diverse crystal structures. This dataset is constructed based on an investigation of various Li-ion cathode materials, covering olivine-type^18^, spinel-type^52^, and layered-type^82^ structures at different Li concentrations, employing the SC DFT + *U* and DFT + *U* + *V* protocols outlined above. Additionally, we include materials such as tunnel- and rutile-type MnO_2_^51,83^ and perovskite-type RE nickelates^53^ in our dataset. The crystal and electronic structure details, magnetic ordering, and various other properties of these materials are extensively documented in their respective publications. These are the compounds containing TM elements Ni, Mn, and Fe, and a list of all these materials is provided in Table 2. While our current dataset is relatively small in terms of material count, ongoing efforts are directed towards a high-throughput exploration involving hundreds of materials, which will serve as an expanded training and validation set for a future, general-purpose, model. This initial dataset demonstrates the efficacy of our ML model, showcasing its ability to predict Hubbard parameters even with limited data (see Table 3). Despite the size of the dataset, the model exhibits accurate predictions, setting the stage for further refinement and expansion with the upcoming comprehensive investigation.

**Table 2:** A list of the materials that are used to train and validate the ML model

Crystal structure type	Chemical composition	#
Olivine	Li_*x*_FePO_4_	5
	Li_*x*_MnPO_4_	5
	Li_*x*_Fe_0.5_Mn_0.5_PO_4_	5
Spinel	Li_*x*_Mn_2_O_4_	2
	Li_*x*_Mn_1.5_Ni_0.5_O_4_	2
Layered	Li_*x*_NiO_2_	2
	Li_*x*_MnO_2_	2
Tunnel	Fe-doped *α*-MnO_2_	1
Tunnel	*α*-MnO_2_	1
Rutile	*β*-MnO_2_	1
Perovskite	YNiO_3_	1
	PrNiO_3_	1
Total	12	28

The last column shows the total number of materials. For the olivine-type materials *x* = 0, 0.25, 0.50, 0.75, 0, while for the spinel- and layered-type materials *x* = 0, 1.

**Table 3:** Chemical elements and the total number of data points for both on-site *U* and inter-site *V* Hubbard parameters determined using the SC protocol illustrated in Fig. 1

Chemical element	Number of data points	
	*U*	*V*
Ni	396 (124)	67,802 (63,332)
Mn	856 (284)	162,272 (153,232)
Fe	138 (120)	22,511 (21,483)
Total	1390 (528)	252,585 (238,047)

These data points are aggregated across all the materials listed in Table 2 that contain the respective chemical element. Numbers in brackets indicate the unique data points after de-duplication (see text).

For each material in our dataset, SC Hubbard *U* and *V* parameters are computed using the SC protocol illustrated in Fig. 1. We include all *V* parameters for Hubbard-active atom pairs whose DFPT values are greater than 0.3 eV, in constrast to ref. ^64^ where only *V* for the nearest neighbor couples were included in the training of their ML model. Throughout the iterative process, all intermediate converged occupation matrices and Hubbard parameters are systematically saved. The dataset for training *V* comprises approximately seven times more data points than that for *U* since *V* is an atom-pair quantity, in contrast to the local and consequently sparser nature of *U*. For each material and chemical element, this data is randomly split, holding back 20% from each material for validation.

In the first-principles calculations, certain input Hubbard parameters (*U*_in_ and *V*_in_) yield a specific ground state and occupation matrices, and the response of the system due to a perturbation through DFPT provides corresponding output Hubbard parameters (*U*_out_ and *V*_out_). We use these quantities to predict either the final SC Hubbard parameter values, or the result of a single DFPT calculation to test various aspects of the model (see details for each experiment below). It is worth noting that many of the structures contain symmetry-equivalent sites, and correlations between results from successive SC steps are prevalent. To address this issue, we implement a de-duplication procedure aimed at mitigating potential leakage of identical data from the training to the validation set. This involves calculating a symmetry-invariant distance between all pairs of ENN inputs for each atomic species in each material, which is then used to cluster duplicates based on a specified distance threshold (see Supplementary Section 4). In random train/validate splits, we sample from these clusters rather than individual data points, ensuring that only one example from each cluster is included in the validation set.

### Accuracy of the ML model in predicting the SC Hubbard parameters

**Fig. 3:**
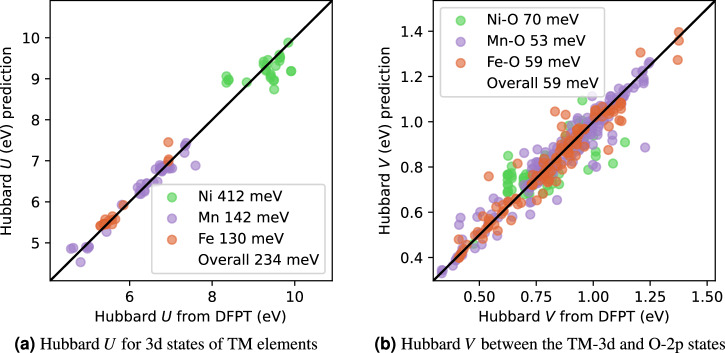
Parity plots showing the prediction accuracy on an unseen validation dataset. **a** Results from Hubbard *U*, and, **b** *V* predictions. The energies in the legend are the RMSE categorized by element(s) and the overall RMSE across all elements. All attributes listed in Table 1 are used as inputs for the ML model.

First, we demonstrate the model’s ability to directly learn the final SC Hubbard parameters for the TM elements across all the materials in our dataset. This is achieved using all attributes listed in Table 1 as inputs for our ML model. Figure 3 shows the parity plots obtained for the on-site *U* and inter-site *V* Hubbard parameters. The mean absolute relative error (MARE) over all species consistently remains below 3% and 5% for the *U* and *V* parameters, respectively (per-species distributions are reported in Sec. S5 in the SI). The result for Hubbard *U* is particularly promising, considering the relatively small amount of available training data. The higher MARE obtained for *V* is likely due to the significantly increased number of degrees of freedom in its model, owing to the involvement of pairs of atoms. It is worth noting that in order to attain this accuracy in predicting the *U* and *V* values, utilizing a relatively small batch size of 8 − 16 was beneficial for preventing overfitting.

Second, we examine the extent to which our ML model depends upon the input Hubbard parameters for its prediction accuracy. To investigate this, we repeat the same numerical experiment as above, eliminating the input Hubbard parameters (*U*_in_ and *V*_in_) from our model. As detailed in Sec. S6 in the SI, the parity plots closely resemble those in Fig. 3. The reduction in accuracy of our ML model resulting from this simplification is relatively small, namely the overall root mean square error (RMSE) is increases by 29 and 18 meV for the on-site *U* and inter-site *V*, respectively. This finding suggests that the input Hubbard parameters may not be the most critical input attributes for our ML model, and that the input occupation matrices contain sufficient information to make low-error predictions.

In addition to evaluating the model accuracy, it is also important to evaluate its impact on downstream properties, such as voltages in Li-ion battery cathode materials. Table 4 shows a comparison of calculated open-circuit voltage (OCV)^18^*Φ* and magnetic moments within the DFT + *U* + *V* framework, using first-principles SC Hubbard parameters from DFPT and those predicted by our ML model. Such OCV have been calculated using standard definitions^84,85^, which, when employed in the present context, imply that the energies of different Li concentrations are evaluated using different Hubbard parameters. This approach was used and discussed in more detail in previous works^18,83,86^, while our goal here is to showcase the consistency between the results obtained with first-principles Hubbard parameters and the ones calculated through the ML model. We find that the differences between the computed and predicted *U* and *V* values are less than 0.12 eV for Li_*x*_MnPO_4_ and Li_*x*_FePO_4_ (*x* = 0 and *x* = 1). Generally, such a small variation in the values of Hubbard parameters has a negligible impact on the vast majority of various physical and chemical properties of materials. The differences observed in the OCV and magnetic moments in Table 4 are indeed negligible, indicating the accuracy and reliability of the ML-predicted Hubbard parameters.

**Table 4:** Comparison of the open-circuit voltages *Φ* (in V) and magnetic moments for TM elements (in *μ*_B_) for Li_*x*_MnPO_4_ and Li_*x*_FePO_4_ computed within DFT + *U* + *V*, with *U* and *V* obtained from first principles using DFPT and predicted using the ML model

*x*	Property	Li_*x*_MnPO_4_	Li_*x*_FePO_4_
0 − 1	*Φ* (DFPT)	4.205	3.544
	*Φ* (ML)	4.194	3.544
	*ΔΦ*	− 0.26%	0.00%
0	*m* (DFPT)	3.9738	4.1828
	*m* (ML)	3.9720	4.1832
	*Δm*	− 0.05%	0.01%
1	*m* (DFPT)	4.7482	3.7388
	*m* (ML)	4.7486	3.7391
	*Δm*	0.01%	0.01%

The voltages are computed using the total energy differences for the Li concentrations *x* = 0 and *x* = 1, while the magnetic moments are computed as the trace of the difference between the spin-up and spin-down occupation matrices^18^. *ΔΦ* and *Δm* are the relative differences between the voltages and magnetic moments based on DFPT and ML, respectively.

### ML model’s performance using a reduced number of iterations in the SC protocol

In the preceding section, we assessed the ML model’s performance in predicting final SC Hubbard parameters using training data that consists of a subset of DFPT calculations from all iterations of the SC protocol (see Fig. 1). However, this approach relies on conducting numerous computationally intensive DFPT calculations to generate these training data (often 2–5 but occasionally up to 10 per material to reach self-consistency).

In this experiment, we instead investigate how well the model can predict the results of a *single* DFPT calculation as a function of how many SC iterations are performed in generating training data. This task is more targeted towards high-throughput applications where data generation for model training could be more efficiently performed by sampling the first few DFPT calculations in the SC workflows of many materials rather than the highly-correlated DFPT calculations of full workflows for a few materials. Effectively, this approach aims to stop the SC cycle early and use a model trained on the calculations already performed to refine the not-yet-SC result to be closer to self-consistency, essentially for free. We investigate this use case by training the model on only the first *N*_iter_ − 1 DFPT results, and asking it to predict the outcome of the next DFPT calculation and study how the model performs for different values of *N*_iter_.

**Fig. 4:**
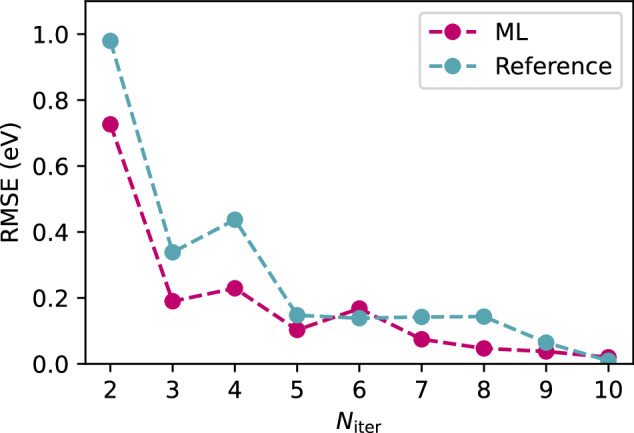
Evaluation of model performance using a reduced number of self-consistent steps. RMSE for all materials as a function of the number of iterations *N*_iter_ in the SC protocol (see Fig. 1). At each *N*_iter_ the plotted reference value is the RMSE between the DFPT result obtained from the previous SC iteration and the current one (i.e., a measure of how much the Hubbard *U* changes by doing one more iteration). The ML value shows the prediction made from having trained on results from all the preceding *N*_iter_ − 1 iterations and predicting the *N*_iter_th value. This helps to answer the question of how much the ML model can improve the Hubbard parameter, at essentially no cost, when limiting the number of DFPT steps as might be done during the high-throughput screening.

Figure 4 shows the average RMSE over all elements as the number of SC iterations used for training *N*_iter_ increases. The reference data correspond to RMSE evaluated using DFPT calculations, i.e., excluding the ML component. This RMSE is computed by determining the difference between the Hubbard parameters at the current *N*_iter_th and the previous (*N*_iter_ − 1)th iteration. It is evident that the reference RMSE decreases non-monotonically, which was observed in previous studies^49^, and eventually diminishes at large *N*_iter_. It is noteworthy that the optimal *N*_iter_ for achieving self-consistency in the SC protocol varies for different materials listed in Table 2. Therefore, for larger *N*_iter_ values in Fig. 4, fewer data points are available compared to smaller *N*_iter_ values.

The ML-based RMSE in Fig. 4 at the *N*_iter_th iteration is computed by training the model on data from all previous *N*_iter_ − 1 iterations and predicting the Hubbard parameters for the *N*_iter_th iteration. Section S7 in the SI contains parity plots for various values of *N*_iter_, ranging from 2 to 7, alongside the reference results broken down by element as well as the results for the training set. It is evident from Fig. 4 that the ML-based RMSE steadily decreases, and for all *N*_iter_ values except *N*_iter_ = 6, it is smaller than the RMSE of the reference dataset. This result shows that our ML model can improve upon a DFPT-based SC result that was terminated early, particularly during the first few iterations, thereby facilitating faster convergence of Hubbard parameters. For instance, the reference value for *N*_iter_ = 2 indicates that on average, a single-shot DFPT calculation yields Hubbard parameters with an RMSE of ~1 eV, while the ML model trained on single-shot data can reduce this error to under 0.8 eV. Incorporating additional *N*_iter_ iterations in the ML training data leads to steady improvements, although the relative performance compared to the reference gradually diminishes until reaching the floor of the model’s accuracy. This floor is partly determined by the convergence of the underlying DFPT-based Hubbard parameters in the SC protocol, conducted with a tolerance of approximately *Δ* = 0.01–0.1 eV (see Fig. 1). Therefore, the most significant computational savings in predicting the SC Hubbard parameters can be achieved using our ML model, which acts as a surrogate, after only a few iterations in the DFPT-based SC protocol. This substantially reduces the overall computational cost and renders it a feasible approach, particularly for high-throughput screening scenarios where a priori training data is unavailable for all materials.

### Transferability of the ML model

To test the transferability of the model, we isolate training data from the olivines (Li_x_*M*PO_4_ with *M* = Fe, Mn, or Fe_0.5_Mn_0.5_) for which we have calculations at Li concentrations *x* = 0, 0.25, 0.5, 0.75, 1. Within this class of materials, the OS of TM elements Mn and Fe changes from +2 to +3 upon delithiation^86^. As mentioned earlier, this change of OS is directly reflected in changes to the occupation matrices^18^. Consequently, the Hubbard parameters for TM elements at different OS also exhibit variation. For instance, the SC Hubbard *U* parameter for Mn changes from 4.56 to 6.26 eV when transitioning from +2 to +3, while for Fe this change is from 5.29 to 5.43 eV^18^. In addition, in Sec. S8 of the SI we present the distribution of Hubbard *U* parameters for Fe and Mn ions in the olivines extracted from the DFPT-based SC protocol for various Li concentrations. Our ML model effectively captures these changes in the Hubbard parameters and accurately predicts their values based on the occupation matrices for each TM ion at different Li concentrations.

**Fig. 5:**
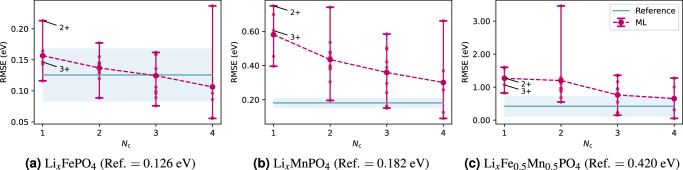
Evaluation of model transferability to unseen electronic structures. **a** Results for Li_*x*_FePO_4_, (**b**) Li_*x*_MnPO_4_, (**c**) Li_*x*_Fe_0.5_Mn_0.5_PO_4_. RMSE as a function of *N*_c_ (the number of Li concentrations) for three olivines. The model is trained using *N*_c_ concentrations and validated on the remaining 5 − *N*_c_. Error bars indicate the range of RMSE values obtained by considering various permutations of concentrations of the training and validation datasets, large dots represent the average, small dots show individual results, and experiments that only contain training data from a single oxidation state are labeled (2+ and 3+). The reference is the RMSE computed by training the ML model on 80% of the Hubbard parameters from all five concentrations, with validation performed on the remaining 20% after de-duplication. The blue line represents the mean results over three runs with different random initializations, the confidence intervals show the standard deviation.

The outcomes of our numerical experiments are shown in Fig. 5. As a reference, we compute the RMSE individually for each material by training the ML model on 80% of the data from all five concentrations and validating it on the remaining 20% of the data after de-duplication. This yields RMSE values of 126, 182, and 420 meV for the Fe, Mn, and mixed Fe-Mn olivines, respectively (see the horizontal lines in Fig. 5). Subsequently, we investigate the RMSE values for scenarios in which the ML model is trained on fewer concentrations, denoted as *N*_c_, to assess the sensitivity of the ML model to the amount of the training data and its transferability for predicting Hubbard parameters at other concentrations. To accomplish this, we train the ML model on *N*_c_ concentrations and validate it on the remaining 5 − *N*_c_ concentrations, and then we average out over all possible permutations of the concentrations between the training and validation datasets. The average RMSE values are represented by larger dots, smaller dots show each individual result, while the error bars in Fig. 5 indicate the maximum and minimum RMSE values resulting from the various permutations. When training on data from a single concentration (*N*_*c*_ = 1) we have labeled the results from *x* = 0 and 1 which correspond to 3+ and 2+ formal OSs respectively, in these cases the model is being tested on OSs it has not seen during training. Four out of six of these give a lower RMSE than experiments where the training data included a mixture of 2/3+ states, suggesting that the model is capable of making meaningful predictions outside of the OS seen during training. More generally, as *N*_c_ increases, the average RMSE for each material decreases, reaching values of approximately 106, 301, and 652 meV at *N*_c_ = 4 for the Fe, Mn, and mixed Fe-Mn olivines, respectively.

To test whether our model can transfer between different crystal structures we perform an additional experiment where we isolate data from the tunnel (*α*) and rutile (*β*) phases of MnO_2_, training on one and testing predictions on the other. Table 5 shows that the model can transfer well between the two phases, achieving RMSEs comparable to those found when training on 80% of the data from both phases and predicting on the rest.

**Table 5:** Hubbard *U* results when training on *α*-MnO_2_ data and predicting on *β*-MnO_2_ and vice versa as well as a reference trained on 80% of all of the deduplicated data and predicting on the remainder

Trained on	RMSE (meV)
80% of *α* & *β*-MnO_2_	144
*α*-MnO_2_	132
*β*-MnO_2_	157

In this case, the model shows good transferability to the unseen crystal structures.

Overall, these experiments demonstrate that, depending on the desired accuracy, it is not necessary to include training data from the exact target system that the model is being evaluated on, and even using examples of one or two similar structures may be sufficient to extrapolate to other compositions or crystal structures. Given the computational cost of SC calculations, this can lead to a significant speedup, particularly in cases requiring larger supercells and various configurations. These findings demonstrate the good transferability of our ML model, indicating its potential utility and reliability for predicting Hubbard parameters in materials not included in the model’s training dataset.

## Discussion

We have introduced a novel equivariant ML model designed for predicting the SC on-site *U* and inter-site *V* Hubbard parameters, thereby circumventing the computationally intensive DFPT-based protocols. The model incorporates three input descriptors: Hubbard parameters, inter-atomic distances, and, notably, atomic occupation matrices. The latter plays a pivotal role in encoding essential information about the electronic structure and local chemical environment within materials. Such an ML model holds significant promise, particularly for high-throughput investigations and large-scale systems, scenarios where DFPT-based approaches become too computationally expensive. Furthermore, the model demonstrates good transferability, rendering it reliable for predicting Hubbard parameters in materials not included in its training dataset, or as a very accurate first guess for further DFPT refinements.

The usage of our ML model is straightforward and entails two DFT-based calculations for a given material. Initially, a ground-state calculation employing DFT + *U* + *V* with initial guesses for *U* and *V* (which can be set to zero) is conducted to determine the atomic occupation matrices required as input for the model. Subsequently, a final structural optimization using the model-predicted SC Hubbard parameters yields a SC structural-electronic ground state. The computational cost of these two calculations and a model evaluation is negligible compared to the DFPT-based SC protocol they replace (see Sec. S10 in the SI). Furthermore, we have demonstrated that the full SC protocol can be reduced to just a few iterations, which prove adequate for achieving a RMSE within a few percent of the real value.

Unlike other ML models designed for predicting Hubbard parameters^27,28,63,64^, our model does not rely on experimental data or information from other state-of-the-art computational methods such as *GW* and hybrid functionals, which possess inherent limitations and specific ranges of applicability. Instead, our model exclusively relies on LR theory through DFPT, which provides material-specific Hubbard parameters directly reflecting the local chemistry and OS of TM elements. Additionally, the current ML model can be trained on different spin configurations of the same material, with these variations reflected in the occupation matrices, though the resulting changes in Hubbard parameters are minor^51,83^. Notably, our model not only predicts on-site Hubbard *U* parameters but also inter-site Hubbard *V* parameters, crucial for materials characterized by significant covalent interactions, an aspect that was disregarded in previous studies (Except ref. ^64^ which is based on the fitting of *U* and *V* to reproduce the results obtained using hybrid functionals, but it does not provide a ML model that can be used to predict the Hubbard parameters for new materials.). Furthermore, the architecture of our ML model is highly versatile, permitting easy integration of additional inputs and outputs, facilitating exploration of diverse learning tasks beyond learning Hubbard parameters. We expect that similar hybrid ML-accelerated electronic structure methodologies, maintaining accuracy and transferability, will become prevalent, potentially yielding a comparable impact on the field as observed with ML interaction potentials.

Lastly, it is essential to highlight the limitations of the trained models we have presented. These were trained on data generated using a specific computational setup (see below), which must remain exactly the same when the model is applied to other systems. In other words, the *U* and *V* values predicted by the model are not transferable across electronic-structure codes and even across different pseudopotentials within the same code^87^. Furthermore, the training data only encompass Fe, Mn, and Ni with Hubbard *U* corrections, while *V* parameters are available for pairs involving these elements and O. Consequently, these models can effectively predict Hubbard parameters for other materials with Hubbard corrections on these atoms or atom pairs, provided that the model input is generated using identical pseudopotentials, Hubbard projectors, and functional (further elaborated in the “methods” section). Nevertheless, with a more diverse dataset, the models could be easily extended to accommodate a broader range of compositions. Indeed, ongoing efforts aim to establish a comprehensive database of Hubbard parameters for various TM-containing materials, akin to ref. ^88^, thereby significantly broadening the scope of our model to encompass numerous TM elements across diverse OS and chemical environments. While we have shown that the models can predict the results of fully SC parameters, models which predict the output of single DFPT calculations may be more applicable in certain situations (see Sec. S11 in the SI). We have shown that models can be trained for this task but have not explicitly tested their performance under recursive evaluation, i.e., performing the SC convergence procedure replacing DFPT completely with an ML model. The performance of the models in this use case and the potential for active learning during this process could be investigated in future work. Moreover, our model can integrate into automated AiiDA workflows^89,90^, enabling non-experts to harness it effortlessly and access SC Hubbard parameters with minimal intervention. Consequently, we believe that our ML model represents a significant advancement in expediting materials discovery, design, and understanding based on the DFT + *U* + *V* approach, thereby unlocking new avenues for technological progress and breakthroughs.

## Methods

### Ab initio calculations

All calculations are performed using the plane-wave pseudopotential method as implemented in the Quantum ESPRESSO distribution^91–93^. We use the exchange-correlation functional constructed using *σ*-GGA with the PBEsol prescription^94^, and the pseudopotentials are taken from the SSSP library v1.1 (efficiency)^95,96^. For metallic ground states, we use Gaussian smearing. To construct the Hubbard projectors, we use atomic orbitals which are orthonormalized using Löwdin’s method^97,98^. Structural optimizations are performed using DFT + *U* + *V*^58^ with the Broyden-Fletcher-Goldfarb-Shanno algorithm^99^ and convergence thresholds for the total energy of 10^−6^ Ry, for forces of 10^−5^ Ry/Bohr, and for pressure of 0.5 Kbar. The DFPT calculations of Hubbard parameters are performed using the HP code^100^, with an accuracy of 0.01–0.1 eV for the computed values of *U* and *V*. The information about the kinetic-energy cutoff, **k** and **q** points sampling of the Brillouin zone for each system are detailed in Sec. S9 in the SI. More technical details can be found directly in the source files publicly available through the Materials Cloud Archive (see below).

## Supplementary information

Supplemental material to the manuscript

## Data Availability

The data used to generate the results presented in this paper are accessible in the Materials Cloud Archive^101^.

## Code availability

The latest version of the code for the equivariant neural networks is available at https://github.com/camml-lab/hubbardml/. Results presented in this paper were generated using version 0.2.0^102^, which is also available on the Materials Cloud Archive^101^.
