# Supplementary material for: Machine learning Hubbard parameters with equivariant neural networks
Source: NPJ Comput Mater. 2025 Jan 25;11(1):19. doi: 10.1038/s41524-024-01501-5 (PMC11761072; doi:10.1038/s41524-024-01501-5)
Supplement: Supplementary file 1 — Supplemental material to the manuscript [file 41524_2024_1501_MOESM1_ESM.pdf]

# Supplemental information for "Machine learning Hubbard parameters with equivariant neural networks"

**Martin Uhrin[1,2,*], Austin Zadoks[1], Luca Binci[1,3,4], Nicola Marzari[1,5], and Iurii Timrov[1,5,+]**

[1]Theory and Simulation of Materials (THEOS), and National Centre for Computational Design and Discovery of Novel Materials (MARVEL), École Polytechnique Fédérale de Lausanne (EPFL), CH-1015 Lausanne, Switzerland
[2]Université Grenoble Alpes, 1130 Rue de la Piscine, BP 75, 38402 St Martin D'Heres, France
[3]Department of Materials Science and Engineering, University of California at Berkeley, Berkeley, California 94720, United States
[4]Materials Sciences Division, Lawrence Berkeley National Laboratory, Berkeley, CA, 94720, USA
[5]Laboratory for Materials Simulations (LMS), Paul Scherrer Institut (PSI), CH-5232 Villigen PSI, Switzerland
*Email: martin.uhrin@grenoble-inp.fr
+Email: iurii.timrov@psi.ch

## S1 Hubbard parameters from DFPT

In Hubbard-corrected DFT, the values of Hubbard parameters are not known *apriori*, making first-principles calculations of Hubbard parameters essential and highly desirable. We compute $U$ and $V$ through a generalized piecewise linearity condition imposed via linear-response theory,[1] based on DFPT.[2,3] Within this framework, the Hubbard parameters are defined as:

$$U^I = \left(\chi_0^{-1} - \chi^{-1}\right)_{II}, \tag{S1}$$

and

$$V^{IJ} = \left(\chi_0^{-1} - \chi^{-1}\right)_{IJ}, \tag{S2}$$

where $\chi_0$ and $\chi$ are the bare and self-consistent susceptibilities, measuring the response of atomic occupations to shifts in the potential acting on individual Hubbard manifolds. $\chi$ is defined as

$$\chi_{IJ} = \sum_{m\sigma} \frac{dn^{I\sigma}_{mm}}{d\alpha^J}, \tag{S3}$$

where $n^{I\sigma}_{mm'} \equiv n^{II\sigma}_{mm'}$ is a short-hand notation for the onsite occupation matrix, $\alpha^J$ is the strength of the perturbation of electronic occupations of the $J$th site, and it is computed at self-consistency of the DFPT calculation. $\chi_0$ has a similar definition, but it is computed before the self-consistent re-adjustment of the Hartree and exchange-correlation potentials.[2] The response of the occupation matrix is computed in a unit cell as:

$$\frac{dn^{I\sigma}_{mm'}}{d\alpha^J} = \frac{1}{N_{\mathbf{q}}} \sum_{\mathbf{q}}^{N_{\mathbf{q}}} e^{i\mathbf{q}\cdot(\mathbf{R}_l - \mathbf{R}_{l'})} \Delta^{s'}_{\mathbf{q}} n^{s\sigma}_{mm'}, \tag{S4}$$

where $\mathbf{q}$ is the wavevector of the monochromatic perturbation, $N_{\mathbf{q}}$ is the total number of perturbations, $\Delta^{s'}_{\mathbf{q}} n^{s\sigma}_{mm'}$ is the lattice-periodic response of atomic occupations to a $\mathbf{q}$-specific monochromatic perturbation. $I \equiv (l,s)$ and $J \equiv (l',s')$, where $s$ and $s'$ are the atomic indices in unit cells while $l$ and $l'$ are the unit cell indices, $\mathbf{R}_l$ and $\mathbf{R}_{l'}$ are the Bravais lattice vectors. The quantities $\Delta^{s'}_{\mathbf{q}} n^{s\sigma}_{mm'}$ are computed from the response Kohn-Sham wavefunctions, obtained by solving $\mathbf{q}$-specific Sternheimer equations. More details about the DFPT approach can be found in Refs. 2,3. The $\mathbf{q}$-point mesh must be chosen dense enough to make the atomic perturbations decoupled from their periodic replicas. It is important to recall that the main advantage of DFPT over the traditional linear-response approach[1] is that it does not require the usage of computationally expensive supercells. Finally, it is crucial to remind that the values of the computed Hubbard parameters strongly depend on the type of Hubbard projector functions $\phi^I_m(\mathbf{r})$ used in the definition of the occupation matrix [see Eq.(3) in the main text] and the Hubbard potential. In this work, we use the atomic orbitals orthogonalized using the Löwdin method.[4,5]

## S2 Self-consistent protocol

Here we explain in more detail the self-consistent protocol for computing Hubbard parameters which is illustrated in Fig. 1(a) in the main text. The process is initiated by the selection of the crystal structure, which can be taken from crystal structure databases, and an initial guess for the input Hubbard parameters, $U_{\text{in}}$ and $V_{\text{in}}$, which can be set to zero. Following this, a structural optimization is performed using DFT+$U_{\text{in}}$+$V_{\text{in}}$, encompassing Hubbard forces and stresses.[6] Subsequently, a DFPT calculation is performed on the relaxed ground state to obtain the output values of Hubbard parameters, $U_{\text{out}}$ and $V_{\text{out}}$. If the input and output Hubbard parameters, as well as the geometry, differ beyond user-specified thresholds, the procedure iterates by updating the input Hubbard parameters and geometry. This iterative cycle continues until convergence is achieved for both the Hubbard parameters and the crystal structure. At the end of this iterative procedure, the final self-consistent (SC) Hubbard parameters, $U_{\text{SC}}$ and $V_{\text{SC}}$, are obtained. These parameters are then used for production calculations using DFT+$U_{\text{SC}}$+$V_{\text{SC}}$. It is worth noting that the SC cycle can take other forms. For instance, one may keep the geometry fixed and converge the Hubbard parameters through multiple DFPT calculations. Only then is the structure updated, followed by the convergence of Hubbard parameters, and this iteration continues until the SC solution is reached. The self-consistent protocol is crucial as it guides the system toward the ground state where the electronic structure and crystal structure are mutually consistent. Additionally, it is important to mention that this protocol can be adapted for use in the DFT+$U$ framework by setting inter-site $V$ to zero.

Figure 1(b) in the main text provides an example of applying the self-consistent protocol to $LiMnPO_4$ in the framework of DFT+$U$.[7] In the first iteration, the Hubbard $U$ parameter for Mn-3$d$ states is approximately 5.08 eV, computed on top of the spin-polarized generalized-gradient approximation ($\sigma$-GGA) (PBEsol)[8] ground state (i.e. $U_{\text{in}} = 0$). However, by the end of the self-consistent protocol, the SC value for $U$ is 4.1 eV — a significant change compared to a "single-shot" calculation (i.e., only the first iteration). The observed change is approximately 1 eV, showing a substantial and impactful adjustment for production calculations. It is important to stress that the output $U$ value after the first iteration strongly depends on the guess of the input $U$ value. Hence, even larger variations may be observed between $U$ values after the first and final iterations. The inter-site $V$ values follow a similar convergence trend.[3] Numerous studies have demonstrated that DFT+$U$+$V$ calculations with SC values for $U$ and $V$ exhibit remarkable agreement with experiments across various materials and properties.[9,10,11,12,13,7,14,15] The self-consistent workflows in our database take on average 4 steps to converge the Hubbard parameters with an accuracy of $\Delta = 0.01 - 0.1$ eV, suitable for the majority of applications.

## S3 Spherical harmonic conventions

To be able to use the occupation matrices as calculated by Quantum ESPRESSO (QE) it is necessary to perform a change of basis from their convention to that used by `e3nn`, the neural network library that we use for generating equivariant learnable functions. The following differences need to be accounted for:

1. QE uses the Condon-Shortley phase convention i.e. the spherical harmonics are defined as

$$Y_\ell^m = \begin{cases} (-1)^m \sqrt{2}\sqrt{\dfrac{2\ell+1}{4\pi}\dfrac{(\ell-|m|)!}{(\ell+|m|)!}}\, P_\ell^{|m|}(\cos\theta)\ \sin(|m|\varphi) & \text{if } m<0 \\ \sqrt{\dfrac{2\ell+1}{4\pi}}\, P_\ell^m(\cos\theta) & \text{if } m=0 \\ (-1)^m \sqrt{2}\sqrt{\dfrac{2\ell+1}{4\pi}\dfrac{(\ell-m)!}{(\ell+m)!}}\, P_\ell^m(\cos\theta)\ \cos(m\varphi) & \text{if } m>0. \end{cases} \tag{S5}$$

   while in `e3nn` the factor of $(-1)^m$ is not absorbed into the definition of $Y_\ell^m$,

2. QE uses the convention that for vectors ($\ell = 1$) $Y_1^{-1} = p_y$, $Y_1^0 = p_z$, $Y_1^1 = p_x$ while `e3nn` uses $Y_1^{-1} = p_x$, $Y_1^0 = p_y$, $Y_1^1 = p_z$, and,

3. QE prints values in the order $m = 0, 1, -1, \ldots, l, -l$ while `e3nn` expects $m = -\ell, -\ell+1, \ldots, 0, \ldots, \ell-1, \ell$.[1]

For the case of $\ell = 1$ the change of basis from QE to `e3nn` is simply the permutation-reflection matrix

$$Q'^{1}_{m'm} = \mathscr{P}^1_{m'm} Q^1_{m'm} = \begin{bmatrix} -1 & 0 & 0 \\ 0 & 1 & 0 \\ 0 & 0 & -1 \end{bmatrix} \begin{bmatrix} 0 & 0 & 1 \\ 1 & 0 & 0 \\ 0 & 1 & 0 \end{bmatrix} \tag{S6}$$

[1] In addition, QE uses column-vector format as opposed to row-vector as used by Python, however this does not affect the occupation matrices as they are symmetric.

However for $\ell > 1$ the situation is more complicated as the spherical harmonics at a given $\ell$ are no longer related by a simple permutation (e.g. $d_{xy}$, $d_{yz}$, $d_z^2$, $d_{xy}$ and $d_{x^2-y^2}$). For these cases, we can use the, so called, Wigner-D matrices:

$$D^l_{m\prime m}(\alpha,\beta,\gamma) = \langle lm'|\hat{R}(\alpha,\beta,\gamma)|lm\rangle \tag{S7}$$

where $\hat{R}(\alpha,\beta,\gamma) = e^{-i\alpha J_z}e^{-i\beta J_y}e^{-i\gamma J_z}$ is the rotation operator parameterised by the three Euler angles. By plugging in $Q_1$ we can get the appropriate rotation matrix at any angular frequency, $Q^\ell = D^\ell_{m'm}(Q_1)$, which can then be pre-multiplied by $\mathscr{P}^\ell = (-1)^m\delta_{m'm}$ to get any $Q'^\ell$. Finally, to make QE's occupation matrices compatible with `e3nn` we simply perform the transformation:

$$n'^\ell_{m'm} = Q'^\ell_{m'm} n^\ell_{m'm} (Q'^\ell_{m'm})^T. \tag{S8}$$

## S4 De-duplication procedure

Our ML model treats each Hubbard active site (or pair of sites) as independent inputs. However, a unit cell may contain more than one symmetry-equivalent site leading to near-identical inputs and outputs. This would bias our statistics when evaluating the performance of the model, particularly if a random split of the data ends up putting two or more near-identical inputs into the training and validation splits, respectively, as the model will be trained on data that it is being validated on. To avoid this, before each training run we de-duplicate the data by finding clusters of near-identical inputs and choosing only one example which then (depending on the nature of the numerical experiment) ends up in either the training or validation set.

To compare permutationally invariant matrices $\mathbf{x}^i_\ell$, we calculate the power spectrum distances between pairs of sites:

$$d^{i,IJ}_\ell = \sum_k \left(\mathbf{x}^i_\ell\right)^I_k \cdot \left(\mathbf{x}^i_\ell\right)^J_k, \tag{S9}$$

where $i$ can be 1 or 2 (see Eqs. (4) and (5) in the main text), $\ell$ is the angular momentum of the manifold, $k$ labels each irrep tensor in the spherical harmonic basis, while $I$ and $J$ label the atomic sites. We calculate $d^{i,IJ}_\ell$ for all pairs of inputs (grouped by species) and form clusters from all data points that are within the thresholds defined in table S1. The similarity threshold for power spectrum distances was chosen by considering the histogram of distances plotted in fig. S1 and choosing a value just above the large peak near zero. This peak should capture the identical electronic occupations with the minimum to its right separating it from the meaningfully distinguishable configurations.

| Attribute | Similarity threshold |
|---|---|
| $U_{\text{in}}$ | $10^{-3}$ eV |
| $V_{\text{in}}$ | $10^{-3}$ eV |
| $d^{1,IJ}_\ell$ | $10^{-4}$ |
| $d^{2,IJ}_\ell$ | $10^{-4}$ |
| $r_{IJ}$ | $4\times10^{-3}$ Å |

**Table S1.** Similarity thresholds for each of the input attributes provided to the ML model. $U_{\text{in}}$ and $V_{\text{in}}$ are the input on-site and inter-site Hubbard parameters, respectively, $\mathbf{x}^1_\ell$ and $\mathbf{x}^2_\ell$ are the tensors defined in Eqs. (5) and (6) in the main text, and $r_{IJ}$ is the interatomic distance between species $I$ and $J$.

## S5 Relative error distributions

Figure S2 shows the results for predicting SC Hubbard parameters relative to the values from DFPT. Taking Hubbard $U$ as an example, this is calculated as $\sum_i^N \left|U^i_{\text{out}} - \tilde{U}^i_{\text{out}}\right| / U_{\text{out}}$, where the sum runs over all $N$ validation results and the predicted value is indicated with a tilde. Given that the range of Hubbard parameters can vary depending on the element, this gives us a consistent way to assess the global performance of the model. For both Hubbard $U$ and $V$ the trend is similar, with Fe and Mn showing similar mean relative errors, while that of Ni is consistently higher. This may be due to the fact that Ni has a relatively wide range of Hubbard parameters (compared to Fe), but we have less training data than for Mn, which also has a much larger spread of self-consistent parameter values.

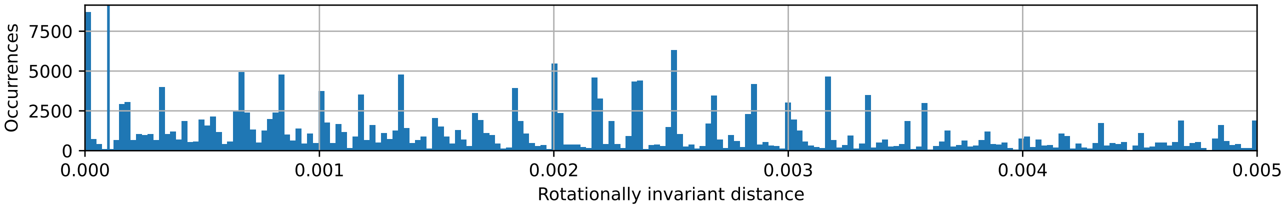


**(a)** Distance analysis for $\mathbf{x}_\ell^1$

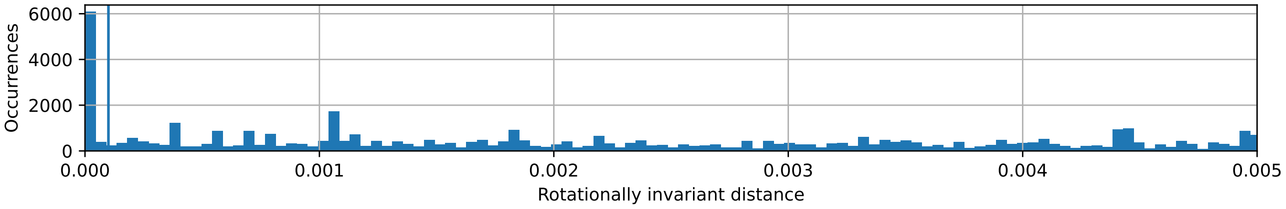


**(b)** Distance analysis for $\mathbf{x}_\ell^2$

**Figure S1.** Analysis of the symmetry-invariant distance distribution for the two permutationally invariant tensors, $\mathbf{x}_\ell^1$ and $\mathbf{x}_\ell^2$. The vertical line represents the chosen threshold for defining two occupation matrices as identical.

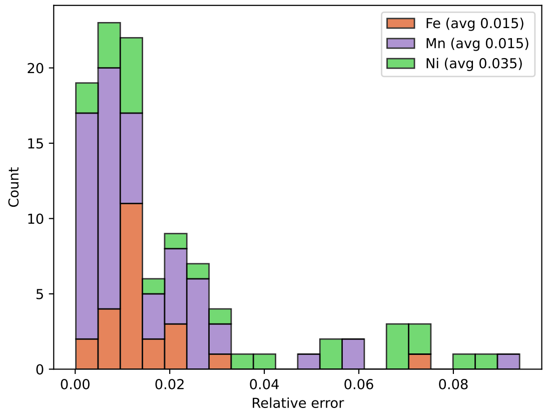


**(a)** Hubbard $U$

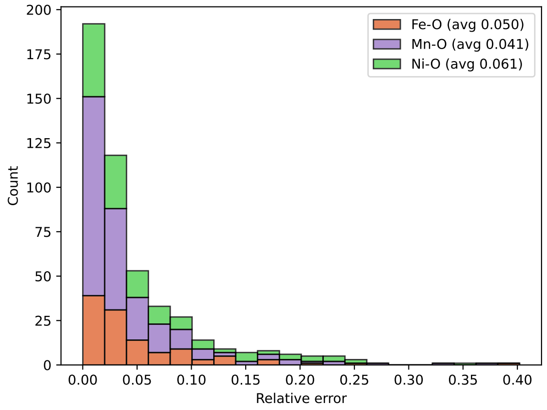


**(b)** Hubbard $V$

**Figure S2.** Validation results for predicting final SC Hubbard parameters plotted as a histogram of errors relative to the values from DFPT.

## S6 ML model without Hubbard parameters as inputs

Figure S3 shows the parity plots using our ML model when excluding the input Hubbard parameters from the list of input attributes. This numerical test demonstrates that the Hubbard parameters may not be the most critical input attributes for our ML model.

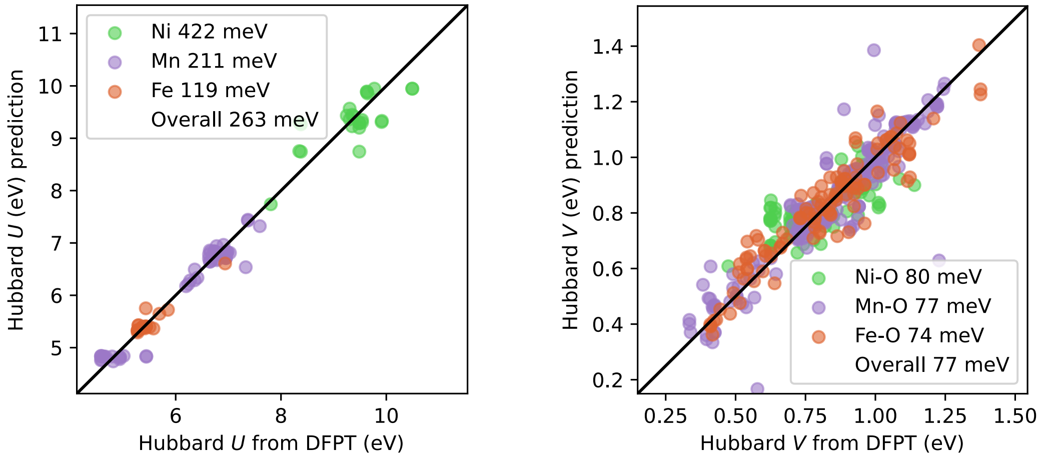


**(a)** Hubbard $U$ (validation) **(b)** Hubbard $V$ (validation)

**Figure S3.** Parity plots showing the prediction accuracy on an unseen validation dataset, where the energies in the legend are the RMSE categorized by element(s) and the overall RMSE across all elements. All attributes listed in Table 1 in the main text (except the input Hubbard parameters) are used as inputs for the ML model. (a) Hubbard $U$ for 3d states of TM elements, (b) Hubbard $V$ between the TM-3d and O-2p states.

## S7 RMSE and parity plots at different number of iterations $N_{\text{iter}}$

Figure S4 show results plotted in fig. 4 of the main article, with the addition of results from the training set. As can be seen, the RMSE on the training results never reach zero as we use early stopping to terminate training when the validation loss starts to increase. In contrast, the validation root-mean-square error (RMSE) gradually at higher $N_{\text{iter}}$ as there are fewer and fewer materials with unconverged Hubbard parameters.

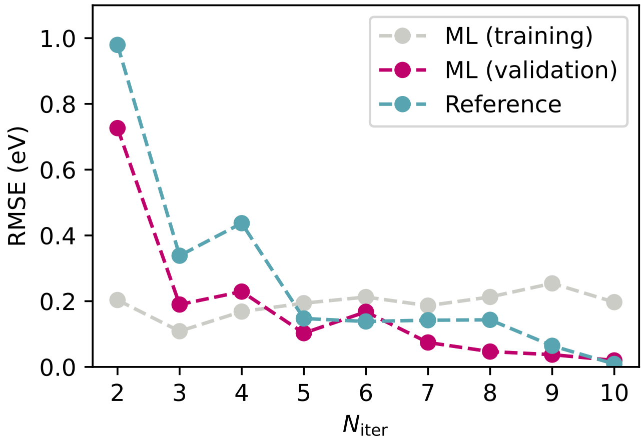


**Figure S4.** RMSE as a function of the number of iterations $N_{\text{iter}}$ in the SC protocol. The reference data represents the RMSE obtained from successive DFPT calculations (without ML involvement), whereas the ML data denotes the RMSE resulting from ML predictions for the $N_{\text{iter}}$th iteration based on training the model on all preceding $N_{\text{iter}} - 1$ iterations, with validation conducted using the DFPT data for the $N_{\text{iter}}$th iteration as a reference.

Shown below are parity plots corresponding to a model that was trained on the first $N_{\text{iter}} - 1$ linear-response calculations,

and asked to predict the Hubbard $U$ value for the current iteration $N_{\mathrm{iter}}$, the "target" is always the actual results from performing the linear-response calculation. The reference plots use the LR result from the previous iteration as the "prediction". In this way, we can assess if the model can learn to improve upon the result obtained from a limited number of DFPT calculations.

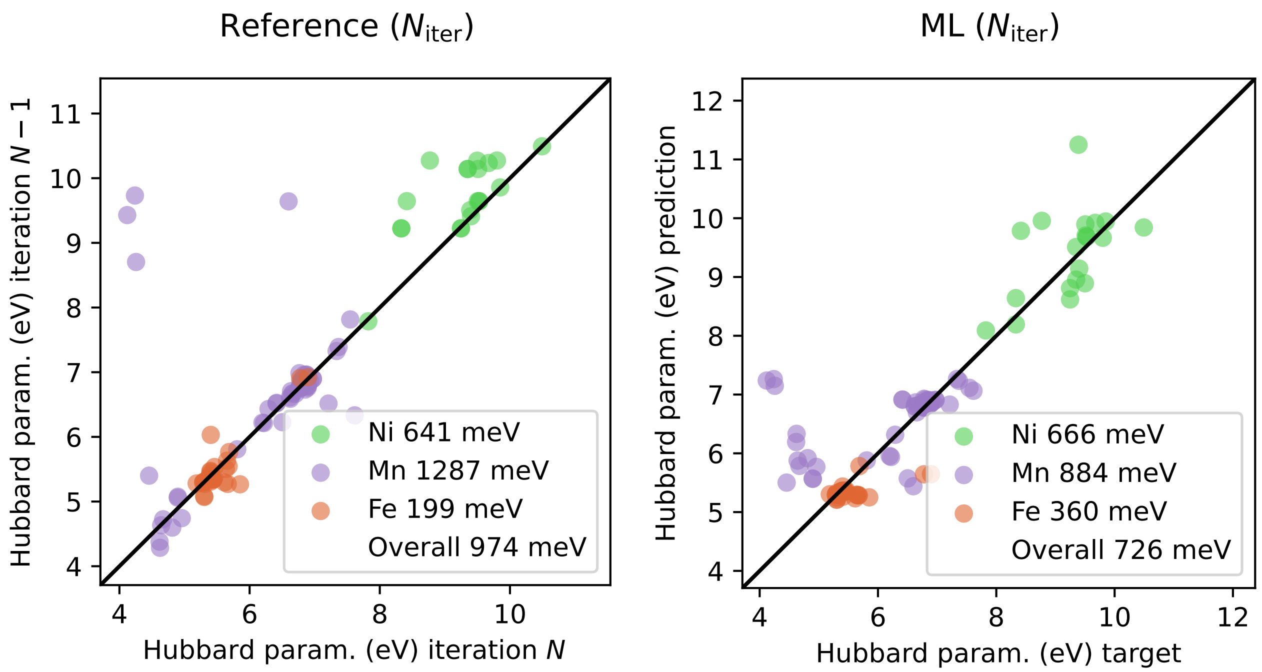


**Figure S5.** Second linear response step.

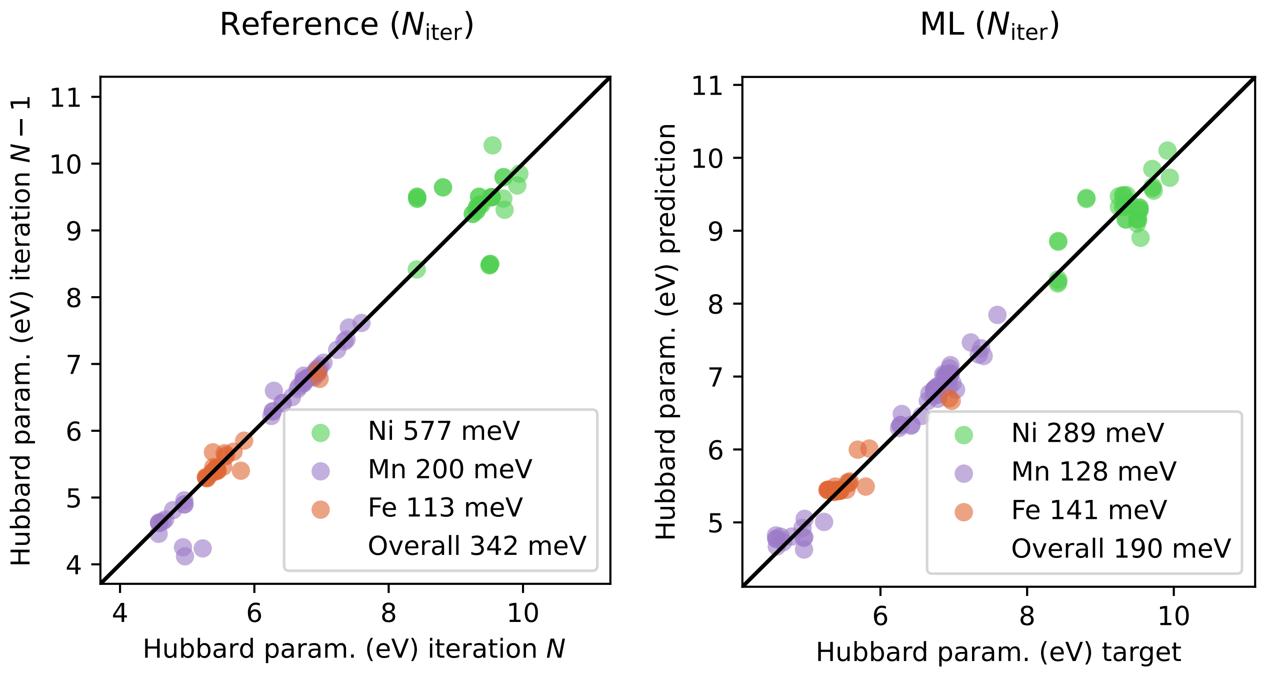


**Figure S6.** Third linear response step.

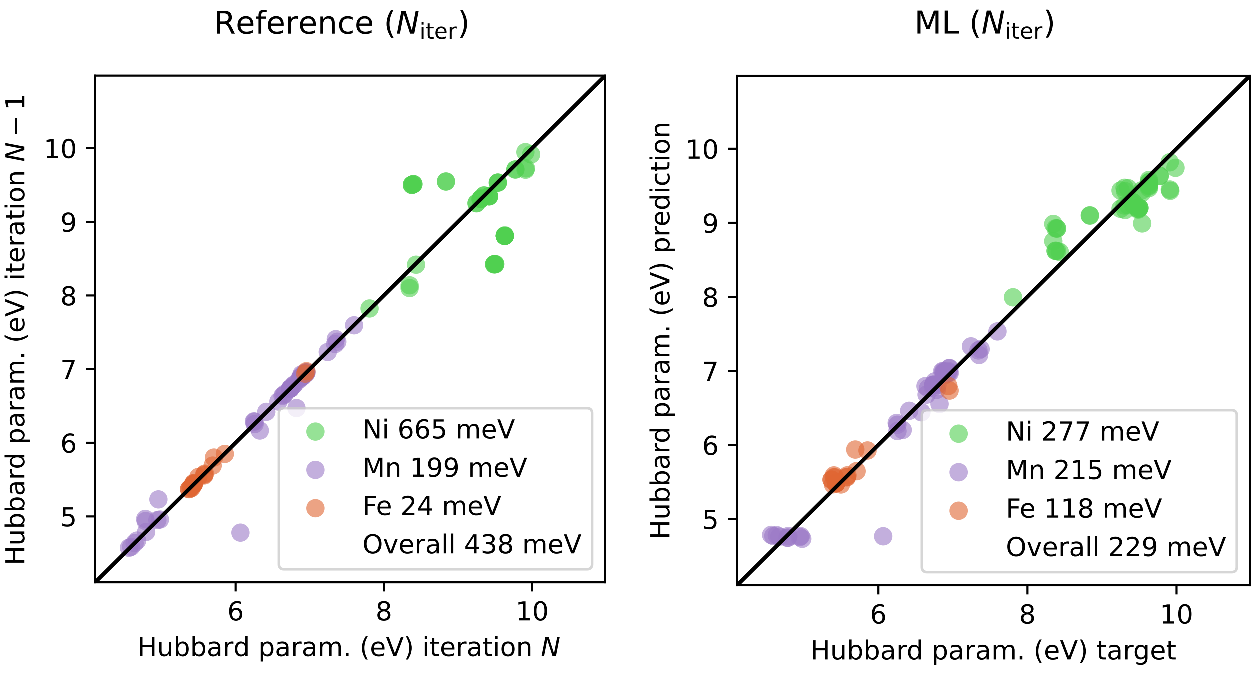


**Figure S7.** Fourth linear response step.

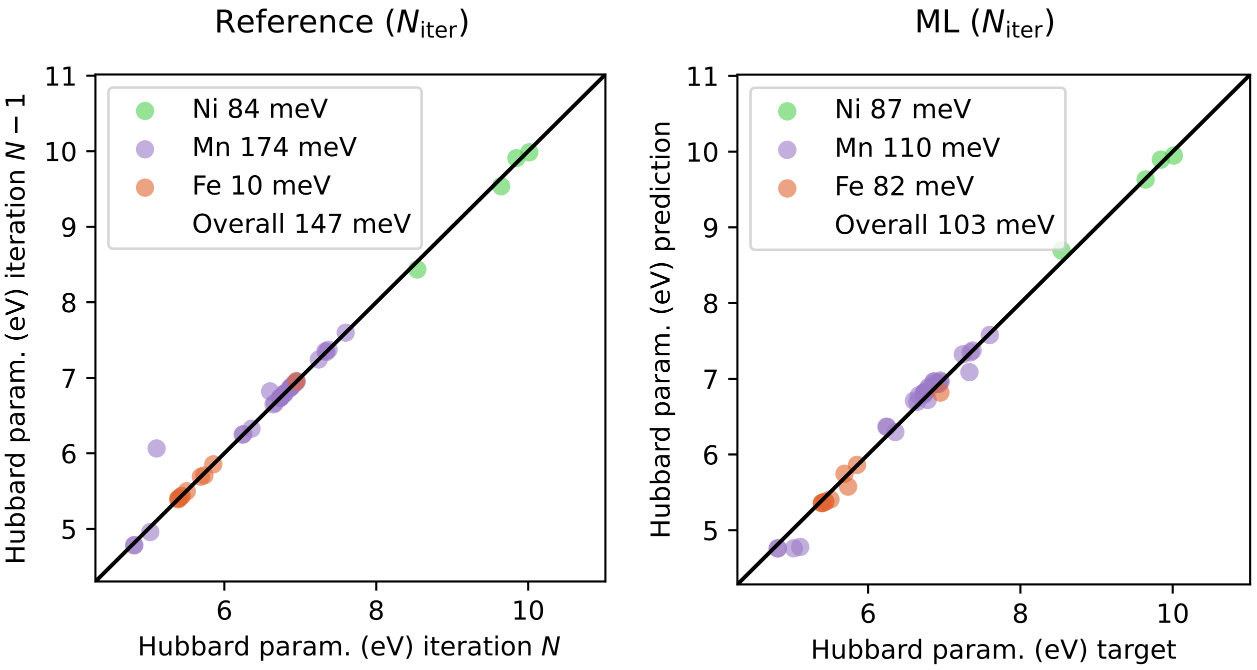


**Figure S8.** Fifth linear response step.

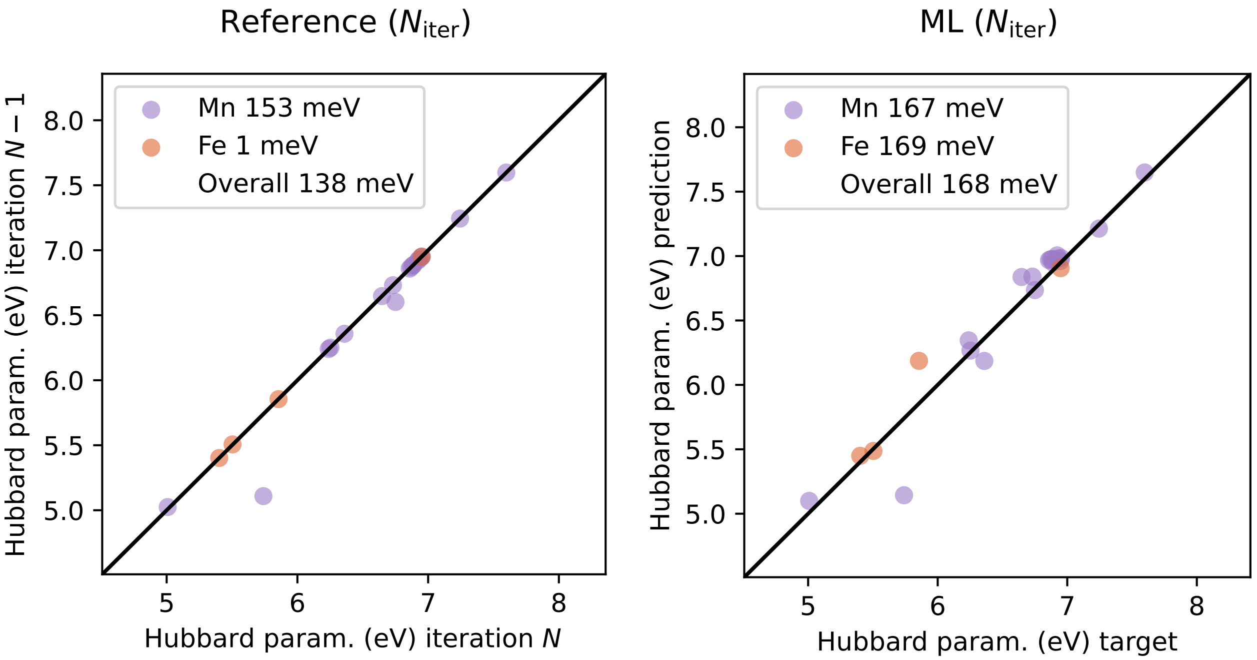


**Figure S9.** Sixth linear response step.

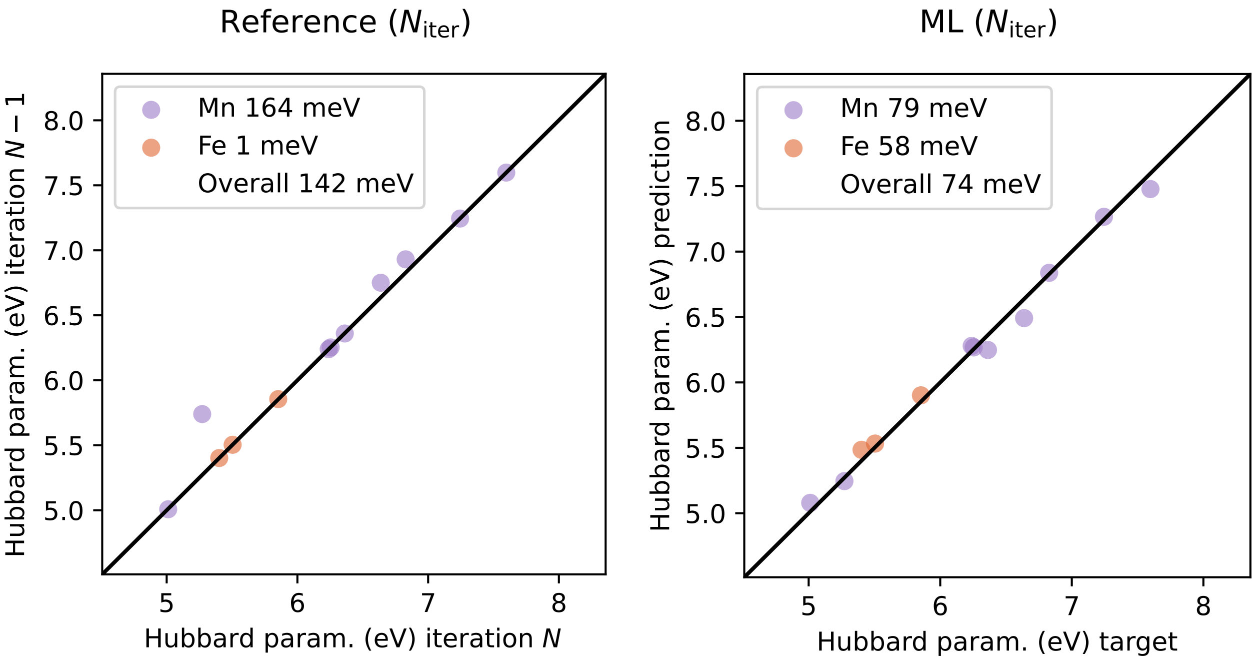


**Figure S10.** Seventh linear response step.

## S8 Olivines linear-response Hubbard $U$ distribution

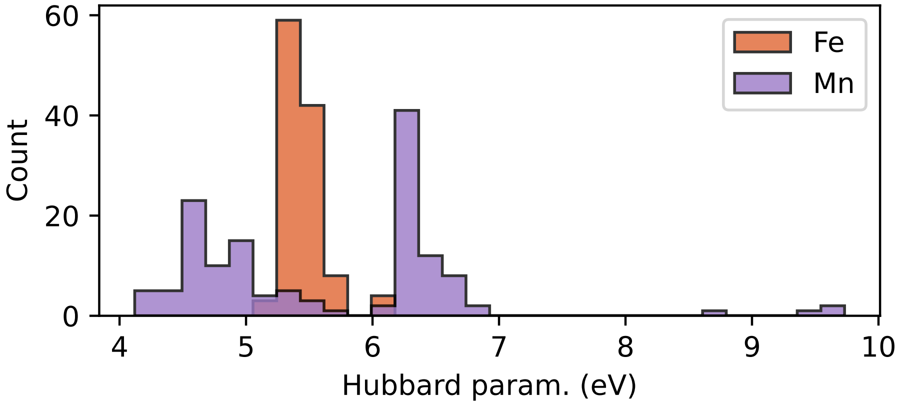


**Figure S11.** Distribution of Hubbard $U$ values obtained from DFPT calculations during a self-consistent procedure. Notably, the distribution of Mn parameters varies significantly more than that of Fe, with two distinct peaks corresponding to the $+2$ and $+3$ oxidation states.

## S9 Additional computational details

| Material | Number of atoms | Structural optimization | | DFPT calculation of Hubbard parameters | | |
|---|---|---|---|---|---|---|
| | | $E_{cut}^{\psi}$ / $E_{cut}^{\rho}$ (Ry) | **k** mesh | $E_{cut}^{\psi}$ / $E_{cut}^{\rho}$ (Ry) | **k** mesh | **q** mesh |
| $Li_xFePO_4$ | 24 – 28 | 90/1080 | $5\times10\times10$ | 65/780 | $3\times4\times5$ | $1\times2\times3$ |
| $Li_xMnPO_4$ | 24 – 28 | 90/1080 | $5\times10\times10$ | 65/780 | $3\times4\times5$ | $1\times2\times3$ |
| $Li_xFe_{0.5}Mn_{0.5}PO_4$ | 24 – 28 | 90/1080 | $5\times10\times10$ | 65/780 | $3\times4\times5$ | $1\times2\times3$ |
| $Li_xMn_2O_4$ | 48 – 56 | 90/1080 | $6\times6\times6$ | 65/780 | $4\times4\times4$ | $2\times2\times2$ |
| $Li_xMn_{1.5}Ni_{0.5}O_4$ | 48 – 56 | 90/1080 | $6\times6\times6$ | 65/780 | $4\times4\times4$ | $2\times2\times2$ |
| $Li_xNiO_2$ | 3 – 4 | 90/1080 | $18\times18\times10$ | 65/780 | $8\times8\times4$ | $4\times4\times2$ |
| $Li_xMnO_2$ | 3 – 4 | 90/1080 | $18\times18\times10$ | 65/780 | $8\times8\times4$ | $4\times4\times2$ |
| $\alpha$-$MnO_2$ | 24 – 25 | 90/1080 | $4\times4\times12$ | 60/720 | $2\times2\times6$ | $1\times1\times3$ |
| $\beta$-$MnO_2$ | 48 | 90/1080 | $4\times4\times6$ | 60/720 | $2\times2\times4$ | $1\times1\times2$ |
| $YNiO_3$ | | | | | | |
| b-type | 40 | 50/400 | $5\times8\times6$ | 50/400 | $5\times8\times6$ | $1\times2\times2$ |
| ferro | 40 | 50/400 | $8\times8\times6$ | 50/400 | $8\times8\times6$ | $2\times2\times2$ |
| s-type | 40 | 50/400 | $5\times8\times6$ | 50/400 | $5\times8\times6$ | $1\times2\times2$ |
| s-type-true | 40 | 50/400 | $5\times8\times6$ | 50/400 | $5\times8\times6$ | $1\times2\times2$ |
| t-type | 40 | 50/400 | $5\times8\times6$ | 50/400 | $5\times8\times6$ | $1\times2\times2$ |
| t-type-true | 40 | 50/400 | $5\times8\times6$ | 50/400 | $5\times8\times6$ | $1\times2\times2$ |
| $PrNiO_3$ | | | | | | |
| b-type | 80 | 50/400 | $4\times8\times3$ | 50/400 | $4\times8\times3$ | $1\times2\times1$ |
| ferro | 20 | 50/400 | $8\times8\times6$ | 50/400 | $8\times8\times6$ | $2\times2\times2$ |
| s-type | 80 | 50/400 | $4\times8\times3$ | 50/400 | $4\times8\times3$ | $1\times2\times1$ |
| t-type | 80 | 50/400 | $4\times8\times3$ | 50/400 | $4\times8\times3$ | $1\times2\times1$ |

**Table S2.** A list of the materials that are used to train and validate the ML model, together with the kinetic-energy cutoff for the wavefunctions ($E_{cut}^{\psi}$) and charge density ($E_{cut}^{\rho}$), the sizes of the **k**- and **q**-point meshes used for the structural optimization and DFPT calculation of Hubbard parameters. Multiple sizes of the **k**- and **q**-point meshes may have been used to accelerate calculations at the beginning of the self-consistent cycle; only the parameters used in the final iteration are reported here.

## S10 Computational cost

An important aspect of computing Hubbard parameters from first principles is computational cost. For illustration purposes, table S3 reports the time cost of the variable-cell relaxation (vc-relax), DFT+$U$+$V$, and DFPT calculations performed in reaching self-consistency of the Hubbard parameters of $Li_{0.25}MnPO_4$. It is worth noting that this timing is highly sensitive to the convergence parameters of the DFT+$U$+$V$ and DFPT calculations among a multitude of other factors; we list this information with the purpose of evaluating the relative cost of the different types of calculations in our highly converged study. For this material, vc-relax and DFPT calculations of the Hubbard parameters require approximately 200$\times$ and 400$\times$ the computational resources of equivalent DFT+$U$+$V$ calculations, respectively. The vc-relax calculation is much more computationally expensive than the DFT+$U$+$V$ ground-state calculation because the former not only requires multiple ionic and cell optimization steps, but also calculations of forces and stresses containing Hubbard contributions. These Hubbard contributions are especially costly when using Löwdin-orthonormalized atomic orbitals as Hubbard projectors[6].

| Iteration | 1 | 2 | 3 | 4 | 5 | 6 | 7 | 8 | Total |
|---|---|---|---|---|---|---|---|---|---|
| vc-relax (node hours) | 236 | 359$^{\dagger}$ | 279 | 229 | 216 | 152 | 105 | 57 | 1,633 |
| DFT+$U$+$V$ (node hours) | 0.85 | 0.99 | 1.00 | 0.87 | 0.93 | 0.94 | 0.91 | 0.98 | 7.47 |
| DFPT (node hours) | 402 | 430 | 389 | 424 | 413 | 419 | 416 | 414 | 3,307 |
| Total (node hours) | 639 | 790 | 669 | 654 | 630 | 572 | 522 | 472 | 4,948 |

**Table S3.** Time cost in node hours for each step of the self-consistent Hubbard workflow for $Li_{0.25}MnPO_4$. $^{\dagger}$The variable-cell relaxation for iteration 2 was stopped early due to non-convergence.

Figure S12 shows a histogram of the DFPT to DFT+$U$+$V$ time ratio for all the SC Hubbard iterations in our dataset, where the median value of the cost multiplier is 114$\times$.

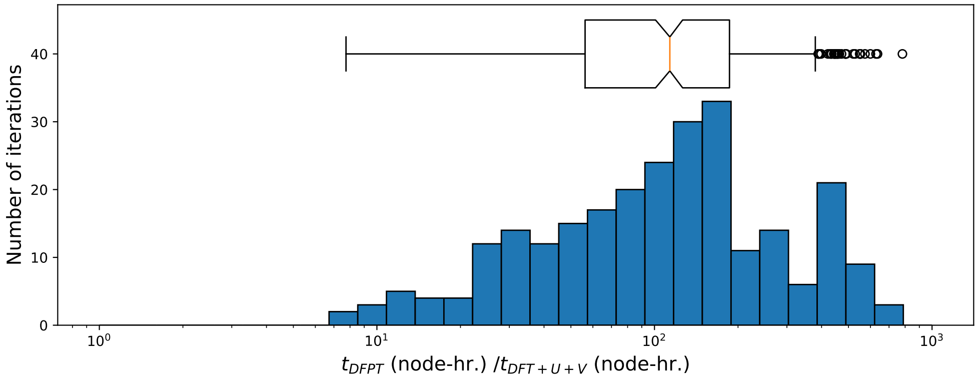


**Figure S12.** Ratio of node-hour time costs between DFPT and DFT+$U$+$V$ calculations per iteration over the dataset. On average, the DFPT calculations cost 2 orders of magnitude more than the DFT+$U$+$V$ calculations (median time ratio of 114).

Figure S13 shows the same histogram comparing vc-relax to DFT+$U$+$V$ calculations, where the median value of the cost multiplier is 50$\times$. Therefore, a median Hubbard SC iteration is expected to cost 165$\times$ that of a DFT+$U$+$V$ calculation of a given material. In order to fully capture the average expected cost of converging the Hubbard parameters for a given material, we also report in fig. S14 a histogram showing the number of iterations required for the 67 systems in our database. The average and median number of iterations are both 4, with a standard deviation of 1.7 iterations. Combining this with the median cost of DFPT and vc-relax calculations, it can be seen that for a median case, the expected cost of convergence is approximately 660$\pm$280 DFT+$U$+$V$ calculations.

In addition to performing DFT+$U$+$V$ and DFPT calculations to generate data, training the ML models is the other potentially significant cost associated to their use in practice. As an example, we report here the training time for the Hubbard $U$ and $V$ models presented in the main text which are trained to predict final self-consistent Hubbard parameters. For this study, the Hubbard $U$ model is trained in 0.85 GPU-hours and the Hubbard $V$ model in 3.73 GPU-hours. At a total time cost of 4.58 GPU-hours, model training is $1-2$ orders of magnitude cheaper (assuming 1 consumer-grade GPU per node) than a single DFPT evaluation for a single material (measured in supercomputer CPU node-hours).

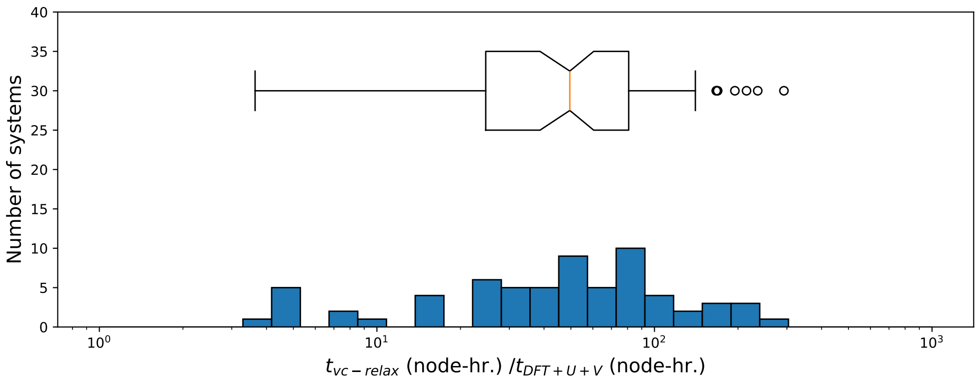


**Figure S13.** Ratio of node-hour time costs between the vc-relax and DFT+$U$+$V$ calculations computed per-system-mean over the dataset. On average, the vc-relax calculations cost one order of magnitude more than the DFT+$U$+$V$ calculations (median time ratio of 50).

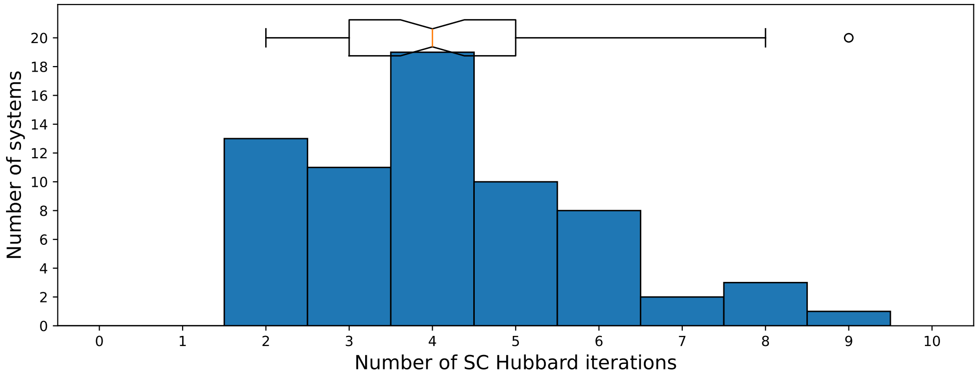


**Figure S14.** Number of self-consistent Hubbard iterations required to achieve convergence. Over the 67 systems represented in the database, the average number of iterations required is 4 with a standard deviation of 1.7.

## S11 Direct substitution of DFPT calculations

In Fig. 3 of the main article we present results from a model that is trained to predict the final, self-consistent Hubbard parameters as we judge this to be the most useful way to use our model. A somewhat easier task is to predict the outputs of a single DFPT calculation, results for which can be seen in fig. S15 when trained on Hubbard $U$ data. Comparing with fig. 3 of the main text, we see that the prediction errors on Mn and Fe are similar while for Ni the results are improved, leading to a lower overall RMSE. By using the model in this mode it is possible to provide a direct surrogate to the DFPT calculation and carry out a self-consistent procedure, however this will incur the higher cost of having to run multiple DFT calculations, for perhaps only modest improvements in the final Hubbard parameters. For those seeking greater accuracy (for example, as a downstream refinement step in a high-throughput study), it is likely to be more beneficial to simply use a self-consistent Hubbard parameter prediction followed by a DFT and DFPT calculations to get a refined result.

## References


1. Cococcioni, M. & de Gironcoli, S. Linear response approach to the calculation of the effective interaction parameters in the LDA+U method. *Phys. Rev. B* **71**, 035105, DOI: 10.1103/PhysRevB.71.035105 (2005).
2. Timrov, I., Marzari, N. & Cococcioni, M. Hubbard parameters from density-functional perturbation theory. *Phys. Rev. B* **98**, 085127, DOI: 10.1103/PhysRevB.98.085127 (2018).
3. Timrov, I., Marzari, N. & Cococcioni, M. Self-consistent Hubbard parameters from density-functional perturbation theory in the ultrasoft and projector-augmented wave formulations. *Phys. Rev. B* **103**, 045141, DOI: 10.1103/PhysRevB.103.045141 (2021).

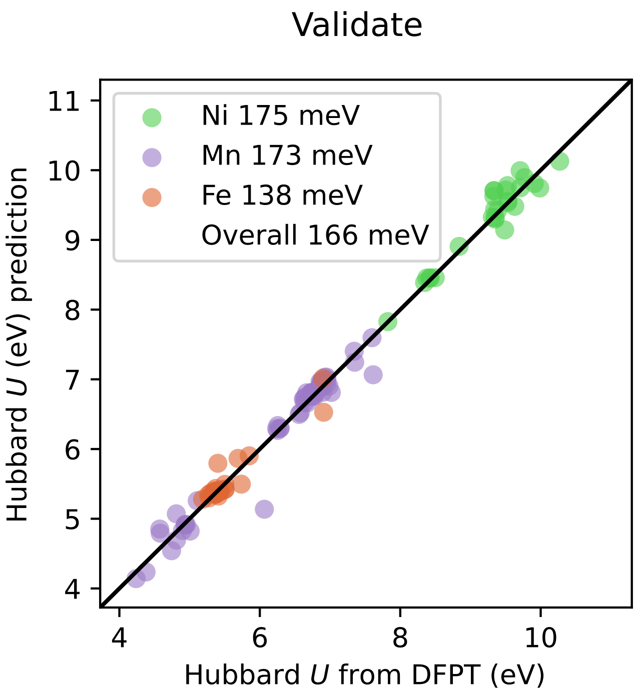


**Figure S15.** Parity plot showing the prediction accuracy on an unseen validation dataset when predicting the output of a DFPT calculation.


4. Löwdin, P.-O. On the Non-Orthogonality Problem Connected with the Use of Atomic Wave Functions in the Theory of Molecules and Crystals. *J. Chem. Phys.* **18**, 365, DOI: 10.1063/1.1747632 (1950).
5. Mayer, I. On Löwdin's method of symmetric orthogonalization. *Int. J. Quant. Chem.* **90**, 63, DOI: 10.1002/qua.981 (2002).
6. Timrov, I., Aquilante, F., Binci, L., Cococcioni, M. & Marzari, N. Pulay forces in density-functional theory with extended Hubbard functionals: from nonorthogonalized to orthogonalized manifolds. *Phys. Rev. B* **102**, 235159, DOI: 10.1103/PhysRevB.102.235159 (2020).
7. Timrov, I., Aquilante, F., Cococcioni, M. & Marzari, N. Accurate Electronic Properties and Intercalation Voltages of Olivine-type Li-ion Cathode Materials from Extended Hubbard Functionals. *PRX Energy* **1**, 033003, DOI: 10.1103/PRXEnergy.1.033003 (2022).
8. Perdew, J. *et al.* Restoring the Density-Gradient Expansion for Exchange in Solids and Surfaces. *Phys. Rev. Lett.* **100**, 136406, DOI: 10.1103/PhysRevLett.100.136406 (2008).
9. Ricca, C., Timrov, I., Cococcioni, M., Marzari, N. & Aschauer, U. Self-consistent DFT+$U$+$V$ study of oxygen vacancies in $SrTiO_3$. *Phys. Rev. Res.* **2**, 023313, DOI: 10.1103/PhysRevResearch.2.023313 (2020).
10. Floris, A. *et al.* Hubbard-corrected density functional perturbation theory with ultrasoft pseudopotentials. *Phys. Rev. B* **101**, 064305, DOI: 10.1103/PhysRevB.101.064305 (2020).
11. Mahajan, R., Timrov, I., Marzari, N. & Kashyap, A. Importance of intersite Hubbard interactions in $\beta$-$MnO_2$: A first-principles DFT+$U$+$V$ study. *Phys. Rev. Mater.* **5**, 104402, DOI: 10.1103/PhysRevMaterials.5.104402 (2021).
12. Zhou, J.-J. *et al. Ab Initio* Electron-Phonon Interactions in Correlated Electron Systems. *Phys. Rev. Lett.* **127**, 126404, DOI: 10.1103/PhysRevLett.127.126404 (2021).
13. Mahajan, R., Kashyap, A. & Timrov, I. Pivotal Role of Intersite Hubbard Interactions in Fe-Doped $\alpha$-$MnO_2$. *J. Phys. Chem. C* **126**, 14353, DOI: https://doi.org/10.1021/acs.jpcc.2c04767 (2022).
14. Timrov, I., Kotiuga, M. & Marzari, N. Unraveling the effects of inter-site Hubbard interactions in spinel Li-ion cathode materials. *Phys. Chem. Chem. Phys.* **25**, 9061, DOI: 10.1039/d3cp00419h (2023).
15. Binci, L., Kotiuga, M., Timrov, I. & Marzari, N. Hybridization driving distortions and multiferroicity in rare-earth nickelates. *Phys. Rev. Res.* **5**, 033146, DOI: 10.1103/PhysRevResearch.5.033146 (2023).
